# Regulation of monocyte cell fate by blood vessels mediated by Notch signalling

**DOI:** 10.1038/ncomms12597

**Published:** 2016-08-31

**Authors:** Jaba Gamrekelashvili, Roberto Giagnorio, Jasmin Jussofie, Oliver Soehnlein, Johan Duchene, Carlos G. Briseño, Saravana K. Ramasamy, Kashyap Krishnasamy, Anne Limbourg, Christine Häger, Tamar Kapanadze, Chieko Ishifune, Rabea Hinkel, Freddy Radtke, Lothar J. Strobl, Ursula Zimber-Strobl, L. Christian Napp, Johann Bauersachs, Hermann Haller, Koji Yasutomo, Christian Kupatt, Kenneth M. Murphy, Ralf H. Adams, Christian Weber, Florian P. Limbourg

**Affiliations:** 1 Department of Nephrology and Hypertension, Hannover Medical School, D 30625 Hannover, Germany; 2 Integrated Research and Treatment Center Transplantation, Hannover Medical School, D 30625 Hannover, Germany; 3 Department of Cardiology, Hannover Medical School, D 30625 Hannover, Germany; 4 Institute for Cardiovascular Prevention, Ludwig-Maximilians-University, D 80336 Munich, Germany; 5 Academic Medical Center, Department of Pathology, Amsterdam University, 1105 AZ Amsterdam, The Netherlands; 6 German Centre for Cardiovascular Research, Partner Site Munich Heart Alliance, Munich, Germany; 7 Department of Pathology and Immunology, Washington University in St Louis, School of Medicine, St Louis, Missouri 63110, USA; 8 Max Planck Institute for Molecular Biomedicine, D 48149 Muenster, Germany; 9 Department of Plastic, Hand and Reconstructive Surgery, Hannover Medical School, D 30625 Hannover, Germany; 10 Department of Immunology and Parasitology, Graduate School of Medicine, Tokushima University, Tokushima 770-8503, Japan; 11 Medizinische Klinik I, Klinikum Rechts der Isar, Technical University of Munich, D 81675 Munich, Germany; 12 Ecole Polytechnique Fédérale de Lausanne, School of Life Sciences, ISREC, CH 1015 Lausanne, Switzerland; 13 Research Unit Gene Vectors, Helmholtz Zentrum München, German Research Center for Environment and Health (GmbH), D 81377 Munich, Germany; 14 Howard Hughes Medical Institute, Washington University in St Louis, School of Medicine, St Louis, Missouri, USA; 15 Cardiovascular Research Institute Maastricht (CARIM), 6229 ER Maastricht, The Netherlands

## Abstract

A population of monocytes, known as Ly6C^lo^ monocytes, patrol blood vessels by crawling along the vascular endothelium. Here we show that endothelial cells control their origin through Notch signalling. Using combinations of conditional genetic deletion strategies and cell-fate tracking experiments we show that Notch2 regulates conversion of Ly6C^hi^ monocytes into Ly6C^lo^ monocytes *in vivo* and *in vitro,* thereby regulating monocyte cell fate under steady-state conditions. This process is controlled by Notch ligand delta-like 1 (Dll1) expressed by a population of endothelial cells that constitute distinct vascular niches in the bone marrow and spleen *in vivo*, while culture on recombinant DLL1 induces monocyte conversion *in vitro*. Thus, blood vessels regulate monocyte conversion, a form of committed myeloid cell fate regulation.

## Abstract (web summary)

Circulating Ly6C^lo^ monocytes are thought to be derived from Ly6C^hi^ subset. Here the authors show that Notch signalling is activated in Ly6C^lo^ cells and is required for their differentiation, and that Notch ligands that initiate this signalling are provided by a subset of endothelial cells.

## Graphical abstract

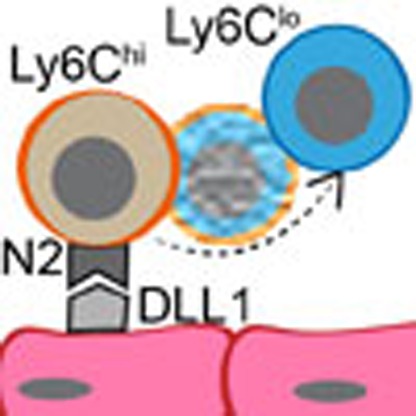

Monocytes are myeloid leukocytes that circulate through blood vessels and patrol the vascular endothelium or differentiate into mononuclear phagocytes. Mouse monocytes consist of two distinct subsets that differ in behaviour and function, which can be discriminated by expression of the Ly6C antigen and CX_3_CR1 chemokine receptor in combination with additional surface markers^1^,^2^. So called classical or Ly6C^hi^ monocytes are characterized by Ly6C^hi^CX_3_CR1^lo^ and constitute the more prevalent subset. They develop from the recently identified common monocyte progenitor (cMoP)^3^, which in turn is derived from the macrophage and dendritic cell progenitor (MDP)^4^. Under steady-state conditions, Ly6C^hi^ monocytes circulate in the blood for short periods of time and are the definitive precursors of certain tissue-resident mononuclear phagocytes, for example in the gut, skin and spleen^2^. When recruited to sites of inflammation through interaction with subsets of activated vascular endothelial cells (ECs), Ly6C^hi^ monocytes give rise to macrophages and dendritic cells, produce inflammatory mediators and orchestrate the inflammatory response^5^,^6^,^7^. Based on functional and gene expression studies, these monocytes correspond to a subset of ‘classical' human monocytes.

There is a second subtype of monocytes that interacts closely and constitutively with blood vessels. They display a Ly6C^lo^CX_3_CR1^hi^ signature and hence are called Ly6C^lo^ monocytes. In the steady-state, Ly6C^lo^ monocytes are long-lived and remain mostly within blood vessels, where they crawl along the luminal side of EC to monitor blood vessels and scavenge microparticles^8^, a feature shared with the human CD16^+^ monocyte subset^9^. After endothelial injury in the kidney, Ly6C^lo^ monocytes orchestrate EC necrosis and clearance^10^. Moreover, Ly6C^lo^ monocytes also mediate IgG-dependent effector functions and are involved in immune complex-mediated disease^11^,^12^. Ly6C^lo^ monocytes were also suggested to contribute to ischemic tissue repair^5^.

The cellular and molecular context of Ly6C^lo^ monocyte development is far from clear. Mice deficient for the transcription factor Nur77 (Nr4a1), an orphan nuclear receptor, show reduced frequency and survival of Ly6C^lo^ monocytes^13^. Recent findings further suggest that in the steady state, Ly6C^lo^ monocytes develop from Ly6C^hi^ monocytes^2^. Grafted MDP and cMoP sequentially give rise to Ly6C^hi^ monocytes followed by Ly6C^lo^ monocytes^3^,^6^. After adoptive transfer of Ly6C^hi^ monocytes, Ly6C^lo^ monocytes are detected in the blood and bone marrow (BM) of recipients, suggesting that Ly6C^hi^ monocytes convert to Ly6C^lo^ monocytes in the circulation^7^,^14^. However, the tissues and molecular events regulating monocyte conversion are unknown.

Notch signalling is a cell to cell contact-dependent signalling pathway regulating cell fate decisions and inflammatory responses in the immune system^15^. Activation of Notch receptors (Notch1–4 in mammals) is controlled by membrane-bound Notch ligands of the jagged (Jag) and Delta-like (Dll) gene families, which show different Notch receptor binding affinities and tissue expression patterns, thereby controlling specific Notch signalling outcomes^16^,^17^. The cellular interactions required for Notch signalling often occur in local tissue microenvironments, or niche, in the BM and spleen^18^. Vascular EC are a specialized component of the niche that maintain and regulate stem cells and their immune cell progeny by providing instructive paracrine cues, known as angiocrine factors, in part through expression of Notch ligands^19^. In the BM niche ECs trigger self-renewal and repopulation of progenitor cells through Notch ligand Jag1 activating Notch1/Notch2 receptors in stem cells^20^,^21^. Similarly, expansion and aggressiveness of B-cell lymphomas is induced by an angiocrine mechanism involving endothelial Jag1 activating Notch2 in malignant lymphoma cells^22^. Specialized vascular niches are also found in secondary lymphoid organs, such as the marginal zone (MZ) of the spleen, which constitutes an EC interface between lymphoid follicles and the red pulp. Development of MZ B cells and Esam^+^ dendritic cell in the splenic niche is dependent on Notch ligand delta-like 1 (Dll1)-Notch2 signalling^23^,^24^,^25^. Monocytes, which are resident in BM and spleen, express Notch1 and Notch2, and their cell fate is influenced by DLL1 *in vitro*^26^.

Because of the intricate relationship of Ly6C^lo^ monocytes with EC we reasoned that blood vessels might be involved in monocyte conversion through a Notch-dependent mechanism. We here show that Notch2 signalling regulates conversion of Ly6C^hi^ monocytes into Ly6C^lo^ monocytes, which is controlled by Notch ligand Dll1 expressed by a population of EC present in haematopoietic niches of the BM and spleen. Thus, blood vessels regulate monocyte conversion, a form of committed myeloid cell fate regulation.

## Results

### Monocyte populations and lineage relationships

**Figure 1:**
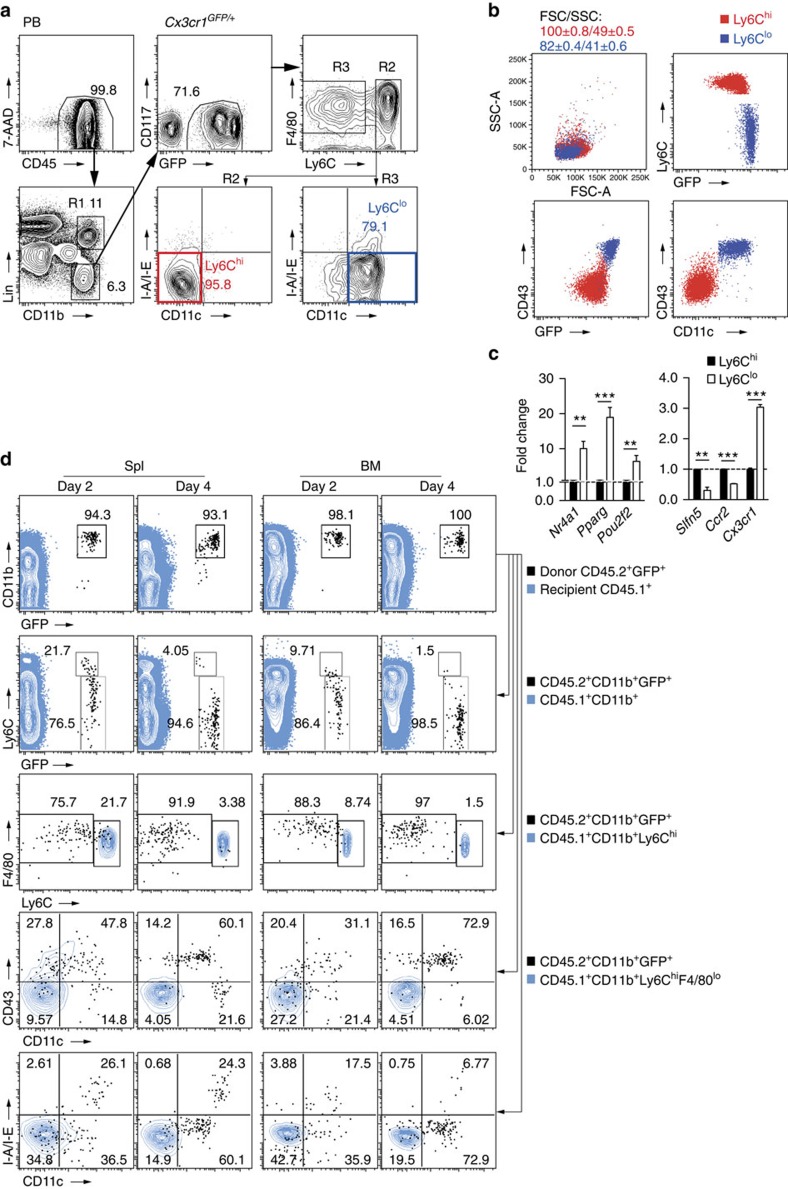
Identification of monocyte subsets and lineage relationships. (**a**) Monocyte subpopulation analysis strategy in PB of *Cx3cr1*^*GFP/+*^ mice. Initially cells were identified based on FSC and SSC characteristics. After exclusion of doublets (on the basis of SSC-W, SSC-A) Lin^−^CD11b^+^ cells were gated from live (7AAD^−^) CD45^+^ gate and CD117^+^ and GFP^−^ populations were excluded. Remaining cells were divided into Ly6C^hi^F4/80^lo/−^ (R2) and Ly6C^lo/−^F4/80^lo^ (R3) subsets. Ly6C^hi^ monocytes were defined from R2 as CD11c^−^MHC-II^lo/−^ (red) and Ly6C^lo^ monocytes from R3—as CD11c^lo^MHC-II^lo/−^ (blue) cells. (**b**) Ly6C^lo^ monocytes are smaller, contain fewer granules than Ly6C^hi^ monocytes and show CD11c^lo^GFP^hi^CD43^+^ phenotype. Numbers are mean±s.e.m. (**c**) Quantitative reverse transcription–PCR analysis performed in sorted monocyte subsets from BM of *Cx3cr1*^*GFP/+*^ mice. Change relative to expression in Ly6C^hi^ cells is shown (*n*=3/6). Error bars represent s.e.m. *P<0.05, **P<0.01, ***P<0.001; Student's *t*-test. (**d**) Dynamics of Ly6C^lo^ monocyte development. BM CD45.2^+^CD11b^+^GFP^+^Ly6C^hi^ monocytes were transferred into CD45.1^+^ recipients and their conversion into Ly6C^lo^ monocytes were followed *in vivo*. Flow cytometry analysis of recipient spleen and BM is depicted. Transferred cells are black and for comparison, recipient CD45.1^+^ (first row), CD45.1^+^CD11b^+^ (second row), CD45.1^+^CD11b^+^Ly6C^hi^ (third row) cells or CD45.1^+^CD11b^+^Ly6C^hi^F4/80^lo^ monocytes (fourth and fifth rows) are shown in blue (representative of two experiments).

Our aim was to study the regulation of Ly6C^lo^ monocytes. To discriminate monocyte subsets and monocyte progenitor populations in mice we concurrently characterized MDP, cMoP, Ly6C^hi^ and Ly6C^lo^ monocytes in BM, spleen and peripheral blood (PB) with common and discriminating markers of monocyte types based on known expression profiles^1^,^2^,^3^,^27^. This approach was tested in *Cx3cr1*^*GFP/+*^ reporter mice^28^, in which monocyte subsets express distinct intensities of green fluorescent protein (GFP), but also in wild-type mice (Fig. 1a, Supplementary Fig. 1a,b and Supplementary Table 1). In addition, monocyte subpopulations were also validated in *Nr4a1-GFP* reporter mice^29^, which demonstrated selective GFP expression in Ly6C^lo^ monocytes (Supplementary Fig. 2a,b). These studies confirmed the proto-typical flow cytometry and gene expression profiles reported for Ly6C^hi^ and Ly6C^lo^ monocytes (Fig. 1b,c)^3^.

Ly6C^lo^ monocytes are reported to derive from Ly6C^hi^ monocytes under steady-state conditions, based on characteristic changes of two markers, CX_3_CR1 and Ly6C, observed after adoptive transfer of Ly6C^hi^ monocytes^7^,^14^. To confirm and extend these findings we performed adoptive transfer studies with Ly6C^hi^ monocytes that were isolated from CD45.2^+^ *Cx3cr1*^*GFP/+*^ mice and intravenously transferred into CD45.1^+^-recipient mice (Supplementary Fig. 3a,b). Cell fate of donor cells, distinguished from recipient by expression of GFP and congenic CD45, was analysed by flow cytometry 2 and 4 days after transfer in BM and spleen. When analysed by GFP and Ly6C expression, transferred Ly6C^hi^ monocytes progressively and uniformly switched to a Ly6C^lo^ monocyte phenotype displaying upregulation of GFP and downregulation of Ly6C (Fig. 1d, Supplementary Fig. 3c). An extended marker analysis demonstrated more complex phenotypic changes involving the progressive acquisition of CD11c and CD43 while maintaining low expression levels of major histocompatibility complex (MHC)-II, consistent with conversion into Ly6C^lo^ monocytes. These changes occurred over a period of 4 days and were observed in BM and spleen (Fig. 1d). Thus, an extended phenotypic analysis confirms conversion of Ly6C^hi^ monocytes into Ly6C^lo^ monocytes.

### Notch2 regulates Ly6C^lo^ monocytes *in vivo*

**Figure 2:**
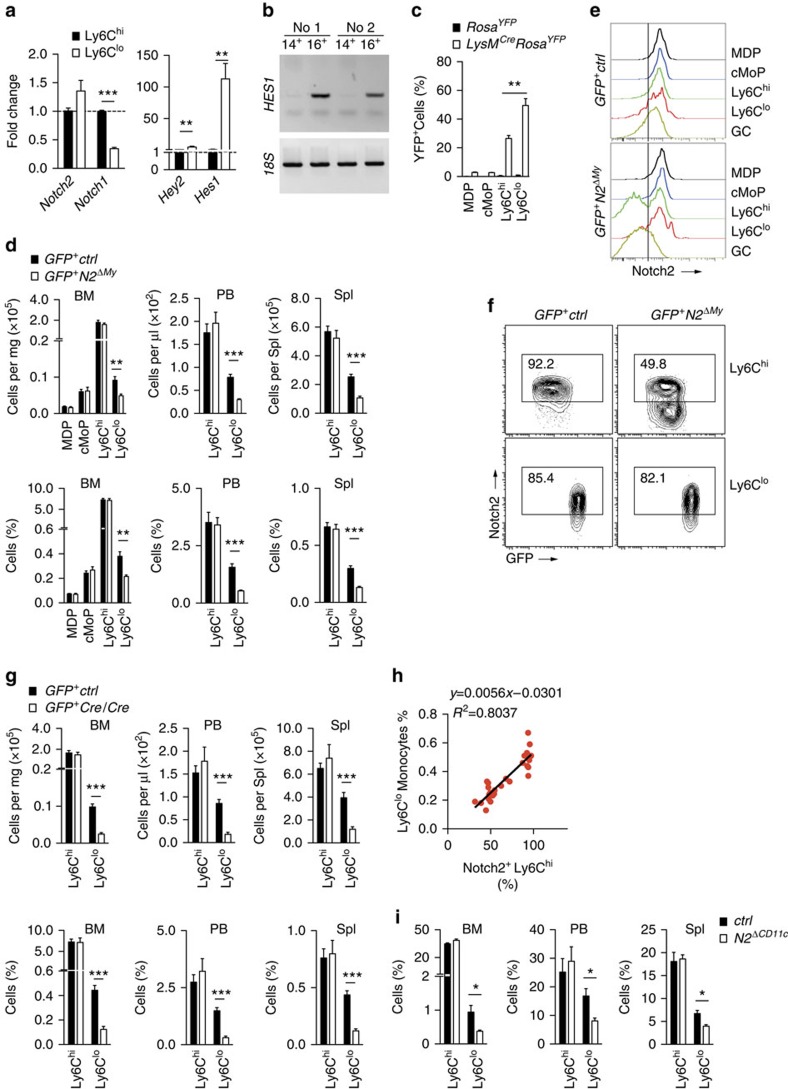
Conditional deletion of *Notch2* impairs Ly6C^lo^ monocyte development. (**a**) Quantitative reverse transcription–PCR analysis in sorted monocyte subsets from BM of *Cx3cr1*^*GFP/+*^ mice; (*n*=6, pooled from three experiments). (**b**) *HES1* expression in human CD14^+^ (classical) or CD16^+^ (non-classical) monocytes from two donors. (**c**) Quantification of YFP^+^ cells in myeloid cells from *LysM*^*Cre*^*Rosa*^*YFP*^ mice as a hallmark of *Cre* activity. Data are pooled from two experiments with three mice in each group. (**d**) Flow cytometry of myeloid cell subpopulations in mice with conditional deletion of *Notch2*. Absolute number of cells per mg BM, per μl blood or per spleen is shown (top). Relative frequency of each subpopulation from live cell gate is shown (bottom). Data are pooled from three experiments with 11/8 mice in each group. (**e**) Notch2 expression in myeloid cell subpopulations from BM of *GFP*^*+*^*Notch2*^*ΔMy*^ mice. (**f**) Notch2 expression in Ly6C^hi^ and Ly6C^lo^ monocyte subpopulations isolated from BM. Littermate controls are shown for comparison. (**g**) Quantification of monocyte subpopulations in mice with conditional deletion of *Notch2* and expressing two alleles of *LysM*^*Cre*^. Absolute number of cells per mg BM, per μl blood or per spleen is shown (top). Relative frequency of each subpopulation from live cell gate is shown (bottom). Data are pooled from three experiments with 12/7 mice in each group. (**h**) Correlation of Notch2^+^Ly6C^hi^ monocyte frequency with frequency of Ly6C^lo^ monocytes. Frequency of Notch2^+^Ly6C^hi^ monocytes shows strong positive correlation with Ly6C^lo^ monocyte numbers (*n*=28). (**i**) Quantification of monocyte subpopulations in *Notch2*^*ΔCD11c*^ mice. Data are pooled from two experiments with four mice in each group. (**a**,**c**,**d**,**g**,**i**) *P<0.05, **P<0.01, ***P<0.001; Student's *t*-test. Error bars represent s.e.m.

To evaluate a potential role of Notch signalling in monocyte subset regulation we sorted Ly6C^hi^ and Ly6C^lo^ monocytes from the BM and analysed Notch-related gene-expression patterns. Compared with Ly6C^hi^ monocytes, Ly6C^lo^ monocytes had lower expression of *Notch1* but comparable *Notch2* expression in messenger RNA and protein (Fig. 2a,e,f). Furthermore, Notch-regulated genes, *Hey2* and *Hes1,* were markedly induced in Ly6C^lo^ monocytes, indicating recent or on-going activation of Notch signalling in this subset (Fig. 2a)^3^,^30^. We next wanted to confirm these findings on corresponding human monocyte subsets. Analysis of the human CD16^+^ monocytes, which are considered equivalents of mouse Ly6C^lo^ monocytes, revealed higher expression of *HES1* compared with the classical CD14^+^ monocytes (Fig. 2b).

We next asked whether Notch deficiency influences monocyte subpopulations. To generate mice with conditional deletions of *Notch* receptors in monocytes we crossed mice bearing floxed alleles of *Notch1*, *Notch2* or *Notch1/Notch2* (refs 17, 31) with a myeloid specific Cre-recombinase strain, *LysM*^*Cre*^ (ref. 32). Strains were also back-crossed onto the *Cx3cr1*^*GFP/+*^ reporter strain (Supplementary Table 2). This targeting strategy was characterized in detail. *LysM* reporter analysis in *LysM-eGFP* mice confirmed low *LysM* promoter activity in progenitor populations, but high promoter activity in Ly6C^hi^ and Ly6C^lo^ monocytes (Supplementary Fig. 4a)^33^. In addition, crossing the *LysM*^*Cre*^ strain to a Cre-dependent YFP reporter strain revealed selective mature myeloid targeting, which was partial in Ly6C^hi^ monocytes, and more efficient for Ly6C^lo^ monocytes and granulocytes (Fig. 2c, Supplementary Fig. 4b), confirming previous reports^34^.

Mice with conditional deletion of *Notch2* (*GFP*^*+*^*N2*^*ΔMy*^) had significantly reduced absolute and relative numbers of Ly6C^lo^ monocytes in BM, PB and spleen (Fig. 2d, Supplementary Fig. 5a). The defect was only observed in Ly6C^lo^ monocytes, since numbers of Ly6C^hi^ monocytes, monocyte progenitor populations and neutrophils were unaffected, and occurred independent of the *Gfp* reporter allele or the *Cre* deleter allele (Fig. 2d and Supplementary Fig. 5b–d). In contrast, mice with conditional deletion of *Notch1* showed no alteration in monocyte subsets (Supplementary Fig. 5e), while combined deletion of *Notch1/Notch2* phenocopied the single *Notch2* mutants (Supplementary Fig. 5f). Altogether, these results demonstrate that monocyte Notch2 controls Ly6C^lo^ monocyte numbers, suggesting a role in monocyte cell fate regulation.

To further investigate the selective reduction of Ly6C^lo^ monocytes we next characterized Notch2 receptor expression by flow cytometry in control mice and conditional mutants. Notch2 was robustly expressed in MDP, cMoP, Ly6C^hi^ and Ly6C^lo^ monocytes in control mice (Fig. 2e,f). In *GFP*^*+*^*N2*^*ΔMy*^ mice, Notch2 expression was not affected in MDPs and cMoP, but substantially reduced in Ly6C^hi^ monocytes, consistent with partial Cre expression and activity in this population (Fig. 2e,f, Supplementary Fig. 4c and (ref. 34)). However, although Cre-mediated targeting is more effective in Ly6C^lo^ monocytes (Fig. 2c, Supplementary Fig. 4b)^34^, most remaining Ly6C^lo^ monocytes in Notch2 mutant mice retained normal Notch2 receptor expression, due to low expression of Cre (Fig. 2e,f and Supplementary Fig. 4c). This suggests that the remaining Ly6C^lo^ monocytes in these mice develop from Notch2-expressing (Cre-negative) Ly6C^hi^ monocytes (Supplementary Fig. 4c). Consistent with this idea, increasing the efficiency of Notch2 deletion in Ly6C^hi^ monocytes, by using *Notch2*^*f/f*^ mice carrying two alleles of *LysM*^*Cre*^, increased the rate of Notch2 deletion in Ly6C^hi^ monocytes but also lead to more strongly reduced levels of Ly6C^lo^ monocytes (Fig. 2g,h), while Ly6C^hi^ monocyte levels remained normal (Fig. 2g). Thus, loss of Notch2 at the level of Ly6C^hi^ monocytes is compatible with generation of Ly6C^hi^ monocytes, but incompatible with generation of Ly6C^lo^ monocytes. Indeed, plotting Notch2 receptor levels on Ly6C monocytes against the frequency of Ly6C^lo^ monocytes revealed that loss of Notch2 on Ly6C^hi^ monocytes correlated strongly with loss of Ly6C^lo^ monocytes (Fig. 2h). This also suggested that the fate of Ly6C^lo^ monocytes is linked to Ly6C^hi^ monocytes through Notch2.

We next wanted to test the effects of a more selective deletion of Notch 2 within the Ly6C^lo^ population. To this end we used a conditional *CD11c-Cre* transgenic approach to delete Notch2 at early stages of Ly6C^lo^ monocyte development, since CD11c is selectively upregulated in Ly6C^lo^ monocytes at early stages of conversion (Fig. 1d, Fig. 5a). Loss of Notch2 partially reduced the number of Ly6C^lo^ monocytes without affecting Ly6C^hi^ monocytes (Fig. 2i, Supplementary Fig. 4d). Altogether, this demonstrates a requirement for Notch2 in the generation and maintenance of Ly6C^lo^ monocytes.

### Physiologic consequences of monocyte Notch2 deficiency

**Figure 3:**
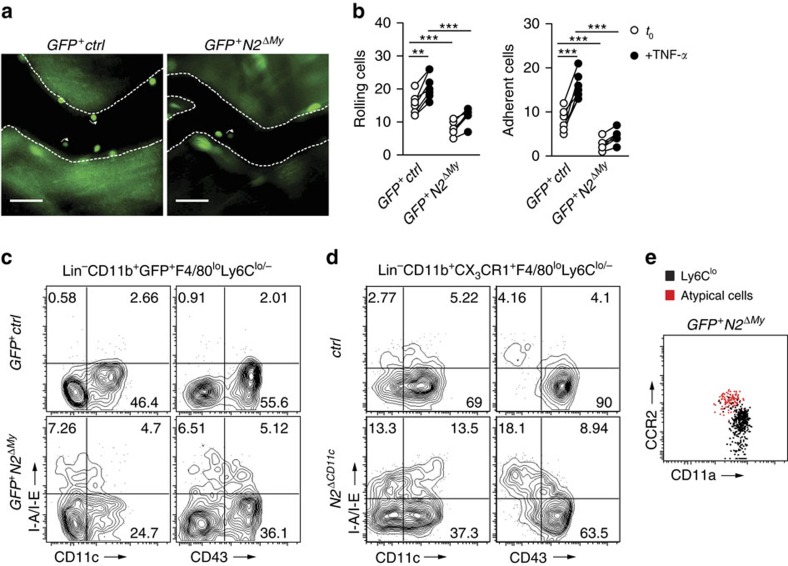
Ly6C^lo^ monocytes show quantitative and phenotypical defects. (**a**) Intravital microscopy image showing GFP^+^ cells in microcirculation of cremaster. Scale bar, 50 μm. (**b**) Quantification of adherent (left) or rolling (right) monocytes per field of view in cremaster blood vessels at *t*=0 or 60 min after infusion of TNF-α. Data are pooled from two experiments (*n*=6/7). ***P<0.001; two-way ANOVA with Bonferroni's post-test. (**c**,**d**) *GFP*^*+*^*Notch2*^*ΔMy*^ (**c**) and *Notch2*^*ΔCD11c*^ (**d**) mice develop an atypical CD11c^−^CD43^−^MHC-II^+^ Ly6C^lo^ population. Representative plots from Lin^−^CD11b^+^GFP^+^F4/80^lo^Ly6C^lo/−^ or Lin^−^CD11b^+^CX_3_CR1^+^F4/80^lo^Ly6C^lo/−^ parental gates, respectively. (**e**) Representative flow cytometry plot showing CCR2 and CD11a expression in Ly6C^lo^ monocytes or atypical cells.

To study the consequences of Notch2 loss of function for monocyte patrolling behaviour in more detail we performed intravital microscopy of the cremaster muscle^8^,^35^. Under steady-state conditions, the number of rolling and adherent Ly6C^lo^ monocytes was greatly reduced in *GFP*^*+*^*N2*^*ΔMy*^ mice, which confirmed the results obtained by flow cytometry (Fig. 3a,b). Furthermore, while TNF-α treatment increased the rolling and adherence of Ly6C^lo^ monocytes in control mice, this response was blunted in *GFP*^*+*^*N2*^*ΔMy*^ mice (Fig. 3a,b), demonstrating a profound impairment of the Ly6C^lo^ monocyte subpopulation with *Notch2* loss of function. This result also ruled out the possibility that mutant Ly6C^lo^ monocytes preferentially localize to blood vessel walls. In addition, the reduction of Ly6C^lo^ monocytes in *Notch2* mutant mice was accompanied by accumulation of an atypical cell population expressing high levels of MHC-II and CCR2 but low levels of CD11c, CD43 and CD11a (Fig. 3c–e), a phenotype not resembling a previously described MHC-II^+^ monocyte population^36^.

### Notch2 regulates Ly6C^hi^ monocyte conversion *in vivo*

**Figure 4:**
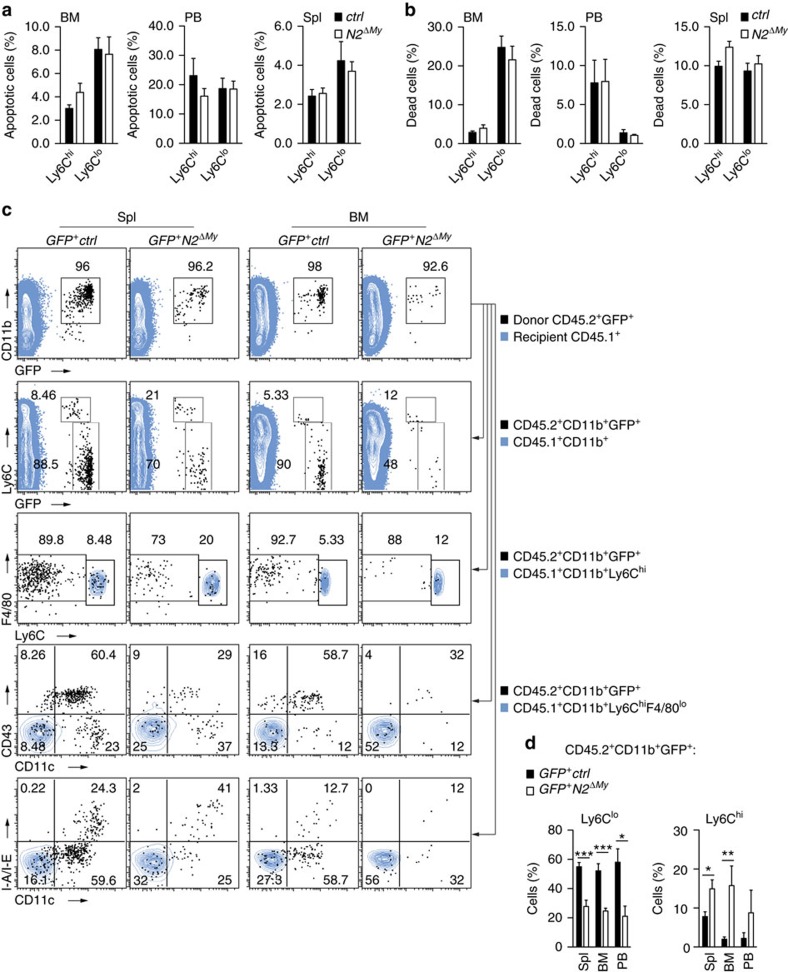
Notch2-deficient Ly6C^hi^ monocytes show impaired conversion potential *in vivo.* Flow-cytometry based quantification of the fraction of (**a**) apoptotic cells (AnnexinV^+^7-AAD^−^) or (**b**) dead cells (7-AAD^+^) in monocyte subsets. Data are pooled from three-independent experiments (*n*=8/12). (**c**) Flow cytometry four days after adoptive transfer of BM Ly6C^hi^ monocytes from control or Notch2-deficient CD45.2^+^GFP^+^ donors into CD45.1^+^ congenic recipients. Transferred cells are shown in black and for comparison, recipient CD45.1^+^ (first row), CD45.1^+^CD11b^+^ (second row), CD45.1^+^CD11b^+^Ly6C^hi^ (third row) cells or CD45.1^+^CD11b^+^Ly6C^hi^F4/80^lo^ monocytes (fourth and fifth rows) are depicted in blue. (**d**) Frequency of donor-derived Ly6C^lo^ monocytes (left) or Ly6C^hi^ monocytes (right) in the CD11b^+^GFP^+^CD45.2^+^ gate after adoptive transfer of Ly6C^hi^ monocytes. Data are pooled from four-independent experiments (*n*=5/7). (**a**,**b**,**d**) *P<0.05, **P<0.01, ***P<0.001; Student's *t*-test. Error bars represent s.e.m.

Ly6C^lo^ monocyte deficiency could be due to increased cell death, as was shown in mice deficient for the transcription factor Nr4a1, which controls Ly6C^lo^ monocyte numbers in part by regulating monocyte apoptosis^13^. We, therefore, tested the hypothesis that Notch2 regulates monocyte survival. However, neither the fraction of apoptotic cells, nor the fraction of dead cells was altered in each of the monocytes subsets in BM, PB or spleen in conditional *Notch2* mutants (Fig. 4a,b).

To address the question whether conversion of Ly6C^hi^ monocytes depends on Notch2 we isolated Ly6C^hi^ monocytes from *Cx3cr1*^*GFP/+*^ control mice or conditional *Notch2* mutants and analysed the cell fate after adoptive transfer into CD45.1^+^ congenic wild-type recipients. Four days after transfer the majority of recovered donor cells from control donors had converted into Ly6C^lo^ monocytes, while few remained Ly6C^hi^ monocytes. After transfer of cells from conditional mutants, however, the fraction of donor cells that had converted into Ly6C^lo^ monocytes was strongly reduced, while the fraction of recovered Ly6C^hi^ monocytes was significantly increased (Fig. 4c,d, Supplementary Fig. 6). Similar results were obtained in experiments when peripheral Ly6C^hi^ monocytes retrieved from PB and spleen were used for transfer (Supplementary Fig. 7), which also ruled out development of Ly6C^lo^ monocytes from contaminating BM precursors. Thus, Ly6C^hi^ monocytes deficient for Notch2 show impaired conversion into Ly6C^lo^ monocytes, demonstrating regulation of monocyte cell fate by Notch2.

### Dll1-Notch2 axis controls monocyte conversion *in vitro*

**Figure 5:**
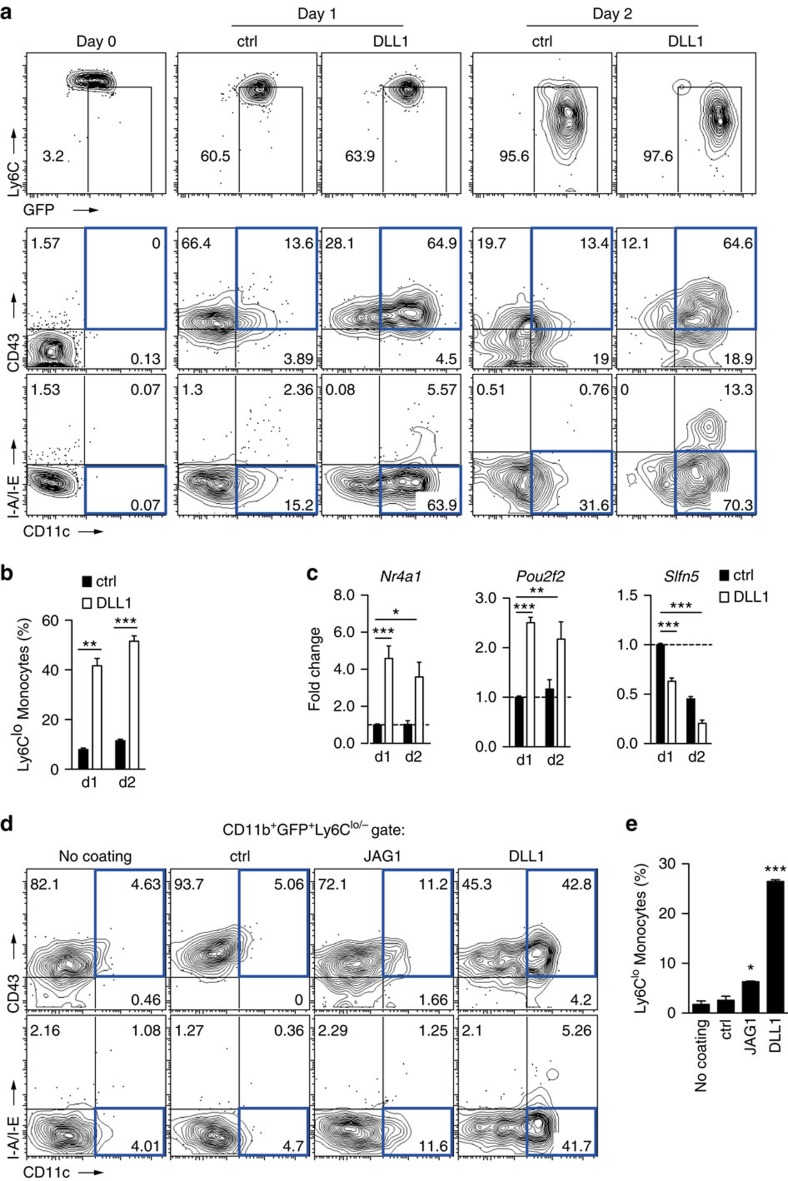
Notch ligand DLL1 mediates monocyte conversion *in vitro*. (**a**,**b**) Kinetics of monocyte conversion in the presence of Notch ligand DLL1 *in vitro*. Representative flow cytometry plots (**a**) and frequency (**b**) of Ly6C^lo^ monocyte-like cells (CD11b^+^GFP^+^Ly6C^lo/−^CD11c^lo^CD43^+^MHC-II^lo/−^) calculated from live CD11b^+^GFP^+^ cells are shown (*n*=3). (**c**) Gene expression analysis in *in vitro* cultures from sorted BM Ly6C^hi^ monocytes. Change relative to expression of genes in control cultures is shown (*n*=6). (**d**,**e**) DLL1 specifically induces conversion *in vitro*. Representative flow cytometry plot (**d**) and frequency (**e**) of Ly6C^lo^ monocyte-like cells (similar to Fig. 5a,b) are shown after culture of Ly6C^hi^ monocytes alone or in the presence of control, JAG1 or DLL1 ligands. (**b**,**c**,**e**) *P<0.05, **P<0.01, ***P<0.001; one-way ANOVA with Bonferroni's multiple comparison test. Error bars represent s.e.m.

Notch receptors are differentially engaged by Notch ligands, and Notch2 is a preferred target of DLL1 (ref. 24). To define the Notch signalling components regulating monocyte conversion, and to provide proof-of-principle that Notch activation is sufficient to regulate this process, we established an *in vitro* culture system to mimic the initial steps during monocyte conversion under defined conditions. We sorted Ly6C^hi^ monocytes from BM of *Cx3cr1*^*GFP/+*^ mice (Fig. 5a day 0) and cultured them in the presence of immobilized recombinant DLL1 protein, or control conditions (Fig. 5a and Supplementary Fig. 8). In culture, downregulation of Ly6C and upregulation of GFP occurred in all conditions over time, while cells remained uniformly CD115^+^ (Fig. 5a, Supplementary Fig. 8b). However, conversion of Ly6C^hi^ into Ly6C^lo^ monocyte-like cells, detected by upregulation of CD43 and CD11c in MHC-II^lo/−^ cells, occurred to a significantly greater extent on DLL1 than in control cultures (Fig. 5a,b). Furthermore, in gene expression profiling, cells cultured on DLL1 showed significantly higher levels of *Nr4a1* and *Pou2f2* and significantly lower levels of *Slfn5* compared with control culture (Fig. 5c), similar to a Ly6C^lo^ monocyte phenotype^3^.

**Figure 6:**
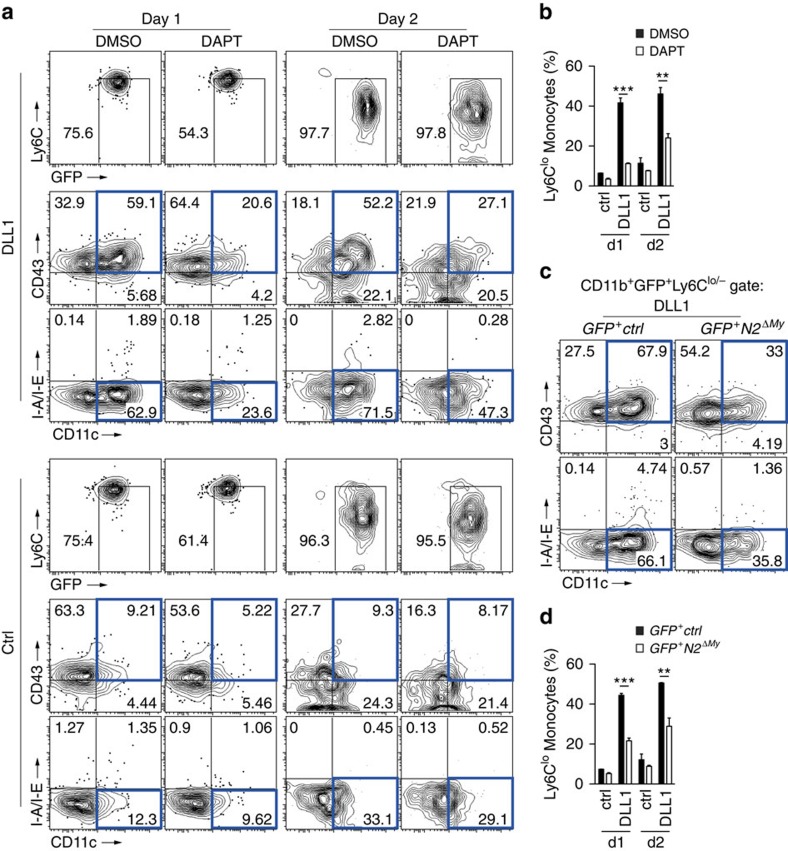
Notch2 signalling is required for monocyte conversion *in vitro*. (**a**,**b**) Inhibition of Notch signalling in sorted Ly6C^hi^ monocytes using a γ-secretase inhibitor (DAPT) impairs conversion *in vitro.* Representative flow cytometry plot from live CD11b^+^GFP^+^ cells (**a**) and relative frequency (**b**) of Ly6C^lo^ monocyte-like cells (CD11b^+^GFP^+^Ly6C^lo/−^CD11c^lo^CD43^+^MHC-II^lo/−^) are shown. (**c**,**d**) Ly6C^hi^ monocytes from *GFP*^*+*^*Notch2*^*ΔMy*^ mice show impaired conversion. Representative flow cytometry plot (**c**) and relative frequency (**d**) of Ly6C^lo^ monocyte-like cells are shown (similar to Fig. 6a,b). (**b**,**d**) *n*=3, *P<0.05, **P<0.01, ***P<0.001; one-way ANOVA with Bonferroni's Multiple comparison test. Error bars represent s.e.m.

The effect was specific for DLL1, since culture of Ly6C^hi^ monocytes on another Notch ligand, JAG1, was much less effective in generating Ly6C^lo^ monocyte-like cells, as were control conditions (Fig. 5d,e). Furthermore, DLL1-induced monocyte conversion was impaired by incubation with a γ-secretase inhibitor, N-(N-(3,5-difluorophenacetyl)-L-alanyl)-S-phenylglycine t-butyl ester (DAPT), which blocks the generation of the active intracellular Notch domain^37^, thus indicating that DLL1-induced Notch receptor cleavage is required for this process (Fig. 6a,b). Importantly, DLL1-induced monocyte conversion was also severely impaired in Ly6C^hi^ monocytes from *Notch2* conditional mutants when compared with controls (Fig. 6c,d). These results demonstrate that a specific Dll1-Notch2 signalling axis controls conversion of Ly6C^hi^ monocytes into Ly6C^lo^ monocyte-like cells.

### Dll1 is expressed in distinct endothelial niches

**Figure 7:**
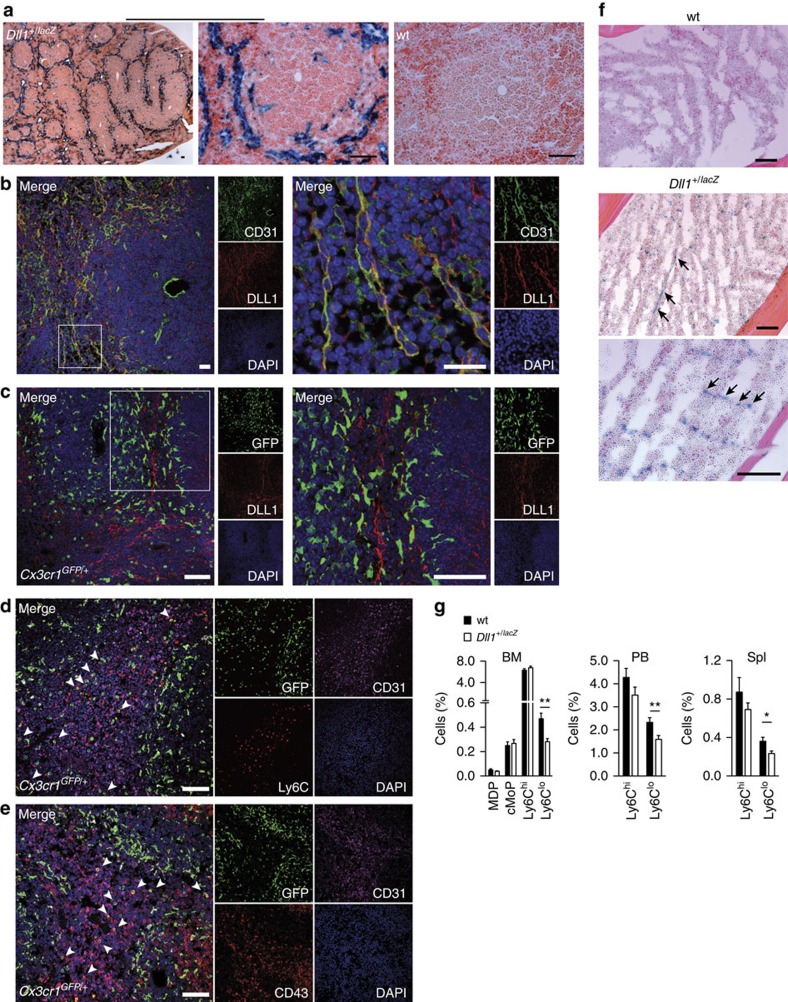
*Dll1* deficiency impairs Ly6C^lo^ monocyte development *in vivo*. (**a**) MZ-specific β-galactosidase activity in splenic follicle of *Dll1*^*+/lacZ*^ mice (left), but not in wt control (right). Scale bars, 50 μm. (**b**) Immunostaining and confocal microscopy of splenic follicle MZ demonstrating DLL1 expression in CD31^+^ EC. Scale bar, 20 μm. (**c**) Immunostaining and confocal microscopy showing GFP^+^ cells in association with DLL1^+^ structures in splenic follicle MZ. Scale bar, 50 μm. (**d**,**e**) Immunostaining and confocal microscopy showing GFP^+^Ly6C^+^ (**d**) or GFP^+^CD43^+^ (**e**) cells in CD31^+^ areas in splenic follicle MZ. Large GFP^+^ cells bordering MZ are Ly6C or CD43 negative. Scale bar, 50 μm. (**f**) *Dll1* expression in BM of femur diaphysis indicated by specific β-galactosidase staining in *Dll1*^*+/lacZ*^ mice; scale bars, 100 μm. (**a**–**f**) representative of more than two experiments. (**g**) Relative numbers of myeloid cell populations by flow cytometry in *Dll1*^*+/lacZ*^ mice or wt littermate controls. Data are pooled from four-independent experiments (*n*=11/12). *P<0.05, **P<0.01, ***P<0.001; Student's *t*-test. Error bars represent s.e.m.

We wanted to corroborate the specific function of Dll1 for Ly6C^lo^ monocyte development *in vivo*. In the adult mouse, Dll1 is selectively expressed in vascular endothelium of arteries, but not veins or capillaries, and in EC in the MZ of the spleen^24^,^37^. We first characterized in more detail Dll1 expression in the two principle haematopoietic compartments, BM and spleen, using genetic reporter mice or immunostaining. In *Dll1*^*+/lacZ*^ reporter mice, in which one allele of *Dll1* has been replaced by *lacZ*, specific reporter staining was observed in the splenic MZ, but not in the central artery of the splenic follicle (Fig. 7a). Immunofluorescence staining against CD31 and DLL1 and confocal microscopy revealed DLL1 expression in EC of the MZ and confirmed its absence in the central artery of the follicle (Fig. 7b). Interestingly, DLL1 staining in *Cx3cr1*^*GFP/+*^ reporter mice demonstrated a close spatial relationship between DLL1 expression and GFP^+^ cell populations in the MZ, suggesting a potential niche function (Fig. 7c). Co-staining with Ly6C or CD43 identified both monocyte subsets within the CD31^+^ and DLL1^+^ MZ area, while large GFP^+^ macrophages reside in the borders of the MZ (Fig. 7d,e and Supplementary Fig. 9a,b). Furthermore, specific Dll1-reporter staining in *Dll1*^*+/lacZ*^ mice was also observed in the BM, which appeared in a reticular pattern in the diaphysal area of the BM cavity, suggesting a vascular pattern (Fig. 7f). More importantly, the defect in Ly6C^lo^ monocytes observed in Notch2 conditional mutants was recapitulated in haploinsufficient *Dll1* mutant mice (Fig. 7g). This demonstrates a critical role for Dll1 in the regulation of Ly6C^lo^ monocytes *in vivo*, and emphasizes the importance of a Dll1-Notch2 axis in the control of Ly6C^lo^ monocyte development. Furthermore, these observations also suggest that distinct Dll1^+^ EC might form specialized niches for the generation of Ly6C^lo^ monocytes.

### Endothelial Dll1 controls Ly6C^lo^ monocyte development

**Figure 8:**
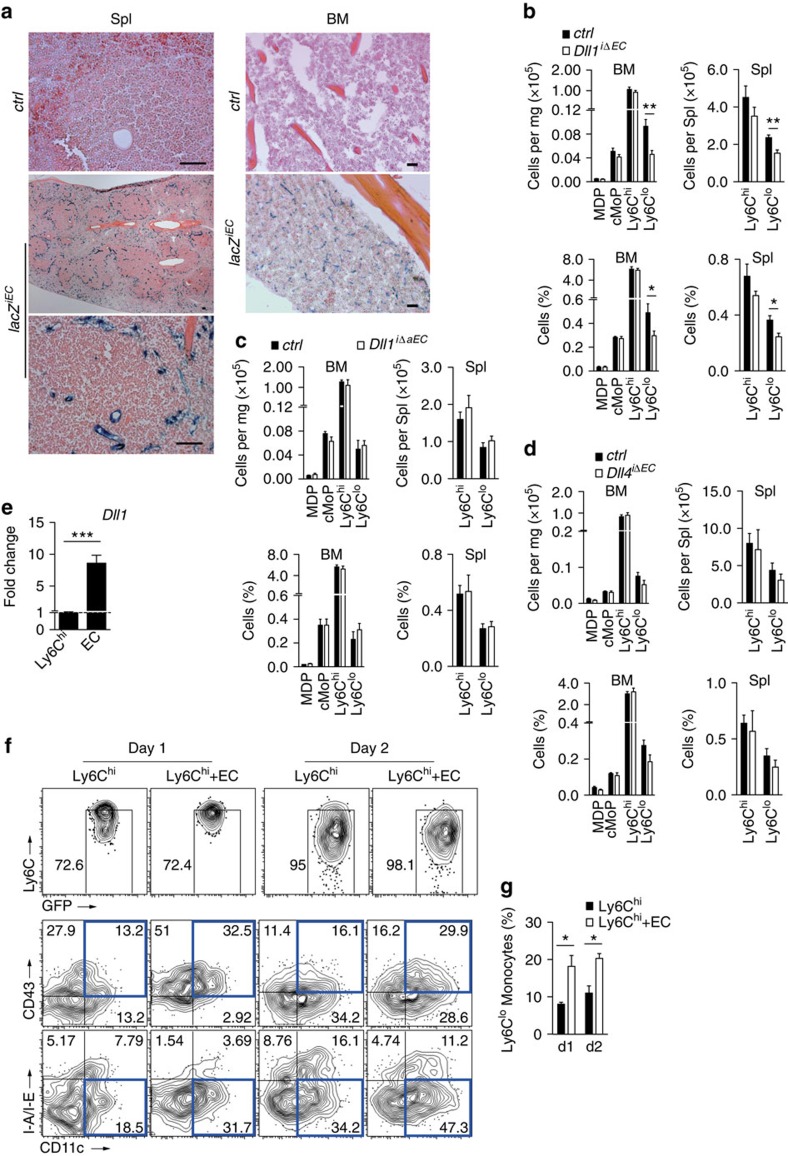
Endothelial deletion of *Dll1* impairs Ly6C^lo^ monocyte development *in vivo.* (**a**) Inducible and endothelial-specific Cre-reporter mice (*lacZ*^*iEC*^) showing specific β-galactosidase staining in splenic MZ (left) and BM (right) of the femur diaphysis after tamoxifen treatment; scale bars, 50 μm. (**b**) Absolute and relative numbers of myeloid cell populations by flow cytometry after pan-endothelial deletion of *Dll1* compared with littermate controls. Data are pooled from four experiments (*n*=8). (**c**) Absolute and relative numbers of myeloid cell populations by flow cytometry after arterial-specific, endothelial deletion of *Dll1* compared with littermate controls. Data are pooled from two experiments (*n*=5/6). (**d**) Absolute and relative numbers of myeloid cell populations by flow cytometry after endothelial-specific deletion of *Dll4* compared with littermate controls. Data are pooled from two experiments (*n*=5). (**e**) Quantitative reverse transcription–PCR analysis of *Dll1* expression in sorted splenic monocytes (CD11b^+^GFP^+^Ly6C^hi^CD144^−^) and ECs (CD11b^−^GFP^−^CD144^+^). Data are pooled from three-independent experiments (*n*=6). (**f**,**g**) Co-culture of splenic CD144^+^ ECs with Ly6C^hi^ monocytes. Representative flow cytometry plot from GFP^+^CD11b^+^ gate (**f**) and frequency of Ly6C^lo^ monocyte-like cells (**g**) are shown (*n*=3). *P<0.05, **P<0.01, ***P<0.001; Student's *t*-test (**b**–**e**) or one-way ANOVA with Bonferroni's multiple comparison test (**g**). (**b**–**e**,**g**) Error bars represent s.e.m.

To test the hypothesis that endothelial Dll1 regulates Ly6C^lo^ monocyte development we employed an endothelial-specific and inducible deletion strategy using the *Cdh5(PAC)-CreERT2* strain, which shows EC-specific Cre activity in peripheral vessels and the BM cavity^16^,^38^. We first confirmed pan-endothelial, but EC-specific, targeting by generating Cre-dependent lacZ-reporter mice (*lacZ*^*iEC*^), which demonstrated inducible EC staining in arteries, veins and capillaries after tamoxifen treatment, but also demonstrated Cre activity in splenic MZ EC and BM EC (Fig. 8a, Supplementary Fig. 9c). Employing a conditional allele of *Dll1* (ref. 23) we next generated endothelial-specific and inducible *Dll1* mutant mice (*Dll1*^*i*Δ*EC*^) and confirmed Cre-dependent recombination and deletion of the conditional *Dll1* allele after a pulse of tamoxifen (Supplementary Fig. 9d,e). In controlled experiments (Supplementary Fig. 7f,g), endothelial deletion of *Dll1* resulted in the selective reduction of Ly6C^lo^ monocytes, while Ly6C^hi^ monocytes and monocyte progenitors where unchanged compared with control (Fig. 8b). These data demonstrate that endothelial Dll1 regulates Ly6C^lo^ monocytes *in vivo*.

Although our data clearly indicated the importance of endothelial Dll1 for monocyte conversion, given the selective expression of Dll1 in two distinct endothelial domains, arteries and the haematopoietic compartment (BM and spleen), the identity of the endothelial domain mediating monocyte conversion remained unclear and could not be addressed with our pan-endothelial deletion strategy. We, therefore, generated mice with inducible, but arterial EC-specific deletion of *Dll1*, by crossing the floxed allele of *Dll1* to *Bmx(PAC)-CreERT2* mice (*Dll1*^*i*Δ*aEC*^)^39^. We confirmed the arterial EC-specific Cre activity in a Cre-dependent lacZ-reporter strain (*lacZ*^*iaEC*^), which demonstrated specific EC staining in aorta, peripheral arteries and central arteries of the splenic follicles, while MZ EC were not targeted, thus providing a tool to address the contribution of arterial EC to monocyte conversion (Supplementary Fig. 10a). We induced *Dll1* deletion in arterial EC, which resulted in Cre-dependent recombination of the conditional *Dll1* allele (Supplementary Fig. 10b). In contrast to pan-endothelial deletion of *Dll1*, arterial EC-specific deletion of *Dll1* did not affect relative or absolute numbers of Ly6C^lo^ monocytes (Fig. 8c), suggesting that Dll1 expressed in endothelial niches in the MZ or BM, and not in arterial endothelium, regulates monocyte conversion.

To further investigate the specificity of Dll1 effects on Ly6C^lo^ monocytes we mated the pan-endothelial and inducible Cre-transgenic strain to conditional alleles of the related Notch ligand *Dll4* (refs 38, 40). In contrast to deletion of *Dll1*, deletion of *Dll4* did not lead to alterations in Ly6C^lo^ monocytes, or any other myeloid subset (Fig. 8d), which demonstrates specific effects of Dll1 in the regulation of Ly6C^lo^ monocytes.

To provide proof-of-principle that EC populations from the haematopoietic compartment regulate monocyte conversion we established an *in vitro* co-culture system. CD144^+^ ECs were sorted from splenic tissue digests and cultured with Ly6C^hi^ monocytes from *Cx3cr1*^*GFP/+*^ reporter mice. We also confirmed Dll1 expression in this EC population (Fig. 8e). Compared with monocytes cultured alone, conversion of Ly6C^hi^ monocytes into Ly6C^lo^ monocyte-like cells was significantly increased on splenic EC, an effect that persisted over time (Fig. 8f,g). Altogether, these results suggest that distinct endothelial niches in the haematopoietic compartments regulate conversion of Ly6C^hi^ monocytes into Ly6C^lo^ monocytes via the Dll1-Notch2 axis.

## Discussion

We here define the molecular and cellular context that regulates monocyte conversion. Taken together, our results show that Notch2 regulates conversion of Ly6C^hi^ monocytes into Ly6C^lo^ monocytes, thereby regulating a specific developmental step in monocyte cell fate under steady-state conditions. This process is controlled specifically by Notch ligand Dll1 expressed by a population of EC that constitute distinct vascular niches in the BM and spleen. Thus, blood vessels regulate monocyte conversion, as a form of developmental cell fate regulation.

The origin and regulation of Ly6C^lo^ monocytes is still poorly understood. Recent findings have suggested that Ly6C^lo^ monocytes develop from Ly6C^hi^ monocytes. Adoptive transfer of cMoP leads to the sequential appearance of Ly6C^hi^ monocytes followed by Ly6C^lo^ monocytes^3^. Furthermore, Jung and colleagues have provided direct evidence that Ly6C^lo^ monocytes derive from Ly6C^hi^ monocytes by adoptive transfer experiments^7^,^14^. Our experiments demonstrating monocyte conversion with isolated Ly6C^hi^ monocytes *in vivo*, and recapitulation of it *in vitro,* clearly support the notion of monocyte conversion as a mechanism regulating development of Ly6C^lo^ monocytes. However, while our data provide evidence for and insights into the mechanism of monocyte conversion, our findings do not exclude the existence of additional mechanisms to generate Ly6C^lo^ monocytes, for example from progenitor cells, as has been recently suggested^41^.

So far, specific molecular or cellular regulators of monocyte conversion have remained elusive, and it has been speculated that monocyte conversion occurs spontaneously in the circulation^2^,^14^. Our data demonstrating a requirement for the Dll1-Notch2 signalling axis for the conversion of Ly6C^hi^ monocytes into Ly6C^lo^ monocytes not only provides clear evidence for the regulated nature of monocyte conversion, but also shows that this process is under control of blood vessels through Notch ligand Dll1. The fact that conditional deletion of Dll4 has no impact on Ly6C^lo^ monocytes further emphasizes the specific nature of Dll1 actions. Although the precise location of monocyte conversion remains unknown, our data clearly demonstrate the existence, and functional importance, of distinct Dll1-expressing vascular niches in BM and spleen. This suggests that monocyte conversion happens, or is at least initiated, in these vascular niches under steady-state conditions. This is further supported by the fact that co-cultured EC from these niches promote monocyte conversion, while our *in vivo* data from mice with arterial-specific *Dll1* deletion exclude the participation of arterial ECs in this process. However, given the dynamic nature of endothelial responses, it is conceivable that in certain situations, such as inflammation, the location, composition and extent of vascular niches might change, which, in turn, might influence monocyte conversion rates.

Recently, the instructive function of the vascular niche for self-renewal and regenerative capacity of HSCs has been defined, which, in part, is mediated by endothelial-specific expression of Notch ligand Jag1 (refs 20, 21). Our finding that monocyte conversion is regulated by ECs extends the spectrum of niche regulation towards more committed steps of myeloid cell development and further supports the importance of the vascular niche in regulating cell fate. On the other hand, the finding that monocyte conversion is specifically regulated by vascular Dll1 underlines the ligand-specific nature of Notch signalling events in different locations^16^.

Our data also suggest that Notch2 expressed in monocytes is directly involved in regulation of Ly6C^lo^ monocyte cell fate in response to Dll1. Several lines of evidence support this conclusion. First, Notch2 was required for monocyte conversion in experiments using isolated Ly6C^hi^ monocytes *in vivo* and *in vitro*. Second, when *Notch2* was targeted on Ly6C^hi^ monocytes *in vivo* we found that the extent of Notch2 loss-of-function in Ly6C^hi^ monocytes is related to the loss of Ly6C^lo^ monocytes, while Ly6C^hi^ monocyte numbers are not affected by *Notch2* deletion. On the other hand, targeting of Notch2 in Ly6C^lo^ monocytes also resulted in selective deletion of Ly6C^lo^ monocytes. Together, this demonstrates a requirement for monocyte Notch2 in the generation, as well as maintenance of Ly6C^lo^ monocytes. Finally, Notch2 loss-of-function lead to the appearance of an atypical monocyte population negative for CD11c and CD43 but expressing high levels of MHC-II and CCR2. These findings suggest a model in which Notch2 regulates Ly6C^lo^ monocyte cell fate: active Notch2 signalling mediates conversion into Ly6C^lo^ monocytes, while defective Notch2 signalling leads to MHC-II^hi^ atypical monocytes. While the role and relevance of this atypical monocyte population observed in conditional mutants is currently unclear, it is important to note that, using different gating strategies and experimental approaches, subpopulations of MHC-II-expressing monocytes have recently been described, which acquire antigen for carriage to lymph nodes^36^.

Currently, the molecular effectors of Notch2 in monocytes are unknown. Interestingly, the transcription factor Nr4a1, an orphan nuclear receptor, regulates the survival of Ly6C^lo^ monocytes^13^. Although a role of Nr4a1 in monocyte conversion has not been investigated, mice with general or BM-restricted inactivation of *Nr4a1* showed reduced Ly6C^lo^ monocytes numbers and increased rates of apoptosis. Although we did not observe a cell death phenotype in *Notch2* mutant mice, we found DLL1-dependent regulation of Nr4a1 *in vitro*. Clearly, the molecular regulation of monocyte conversion requires further study.

Our study also describes the first steps towards an approach to recapitulate monocyte cell fate *ex vivo*. This might provide a setting to study and understand the molecular events driving monocyte conversion under defined conditions.

## Methods

### Mice

Mouse strains used in the study are listed in Supplementary Table 2. *Cx3cr1*^*GFP/+*^ mice^28^ (kindly provided by Steffen Jung), *LysM*^*Cre*^ mice^32^, *Dll1*^*+/lacZ*^ mice^42^ (kindly provided by Achim Gossler), *Notch1*^*lox/lox*^ and *Notch2*^*lox/lox*^ mice^17^, *Cdh5(PAC)-CreERT2* mice^43^, *Bmx(PAC)-CreERT2* mice^44^, *Dll1*^*lox/lox*^ mice^23^*, Dll4*^*lox/lox*^ mice^45^, *N2*^Δ*CD11c*^ mice^46^*, N1N2*^Δ*CD11c*^ mice^47^, *Nr4a1-GFP* mice^29^, *LysM-eGFP* mice^33^*, LysM*^*Cre*^*Rosa*^*YFP*^ mice^48^ have been described. *Gt(ROSA)26Sor* mice carrying Cre-inducible *lacZ* alleles were obtained from The Jackson Laboratories, B6.SJL*-Ptprc*^*a*^*Pepc*^*b*^*/*BoyJ (CD45.1^+^) mice from Charles River. Mice were housed under specific pathogen free conditions in the animal facility of Hannover Medical School unless otherwise indicated. *Nr4a1-GFP, LysM-eGFP* and *LysM*^*Cre*^*Rosa*^*YFP*^ mice were housed in IPEC, Munich, Germany; *N2*^Δ*CD11c*^ mice in WUSTL, St Louis, MO, USA; *N1N2*^Δ*CD11c*^ mice in Tokushima University, Tokushima, Japan and *Cdh5(PAC)-CreERT2 Dll4*^*lox/lox*^ mice in MPI Munster, Germany. All experiments were performed with 8–12-weeks-old mice and age and sex matched littermate controls with approval of the local animal welfare boards (LAVES Lower Saxony, Animal Studies Committee at Washington University in St Louis, Animal Research Committee of Tokushima University, North Rhine Westphalia Animal Ethics Committee and Local Animal Committee of District Government of Upper Bavaria).

### Tissue and cell preparation

For single cell suspension mice were killed and spleen, BM and blood were collected. Erythrocytes were removed by red blood cell lysis buffer (Biolegend) or by density centrifugation using Histopaque 1083 (Sigma-Aldrich). After extensive washing cells were resuspended in PBS containing 10% FCS and 2 mM EDTA kept on ice, stained and used for flow cytometry or for sorting.

### Flow cytometry and cell sorting

Non-specific binding of antibodies by Fc-receptors was blocked with anti-mouse CD16/CD32 (TruStain fcX from Biolegend) in single cell suspensions prepared from spleen, PB or BM. After subsequent washing step cells were labelled with primary and secondary antibodies or streptavidin-fluorochrome conjugates (Supplementary Table 3) and used for flow cytometry analysis (LSR-II, BD Biosciences) or sorting (FACSAria; BD Biosciences or MoFlo XDP; Beckman Coulter). Data were analysed by FlowJo software (Treestar). Initially cells were identified based on forward scatter (FSC) and side scatter (SSC) characteristics. After exclusion of doublets (on the basis of SSC-W, SSC-A), relative frequency of each subpopulation from live cell gate or absolute number of each subset (calculated from live cell gate and normalized on mg BM, μl PB or spleen) were determined and are shown in the graphs as mean±s.e.m., unless otherwise stated.

### *In vitro* conversion studies

Ninety-six-well flat bottom plates were coated at room temperature for 3 h with IgG-Fc, JAG1-Fc or DLL1-Fc ligands (all from R&D) reconstituted in PBS. Sorted BM Ly6C^hi^ monocytes were cultured in coated plates in the presence of M-CSF (10 ng ml^−1^), thrombopoietin (TPO, 20 ng ml^−1^), stem cell factor (SCF, 10 ng ml^−1^), insulin-like growth factor (IGF)-II (20 ng ml^−1^), fibroblast growth factor (FGF)-I (10 ng ml^−1^) (all from Peprotech) and Heparin (25 U ml^−1^) at 37 °C for 24 or 48 h. In experiments where the effect of Notch inhibition on conversion process was assessed, 6 μM of γ-secretase inhibitor, DAPT or dimethylsulphoxide (DMSO) was applied to the cells prior to and 24 h after culture. In separate experiments BM Ly6C^hi^ monocytes were co-cultured with sorted splenic CD144^+^GFP^−^CD11b^−^ EC. One or 2 days after culture, cells were harvested, stained and subjected to flow cytometry. Frequency of Ly6C^lo^ monocyte-like cells (CD11b^+^GFP^+^Ly6C^lo/−^CD11c^lo^MHC-II^lo/−^CD43^+^) in total live CD11b^+^GFP^+^ cells served as an indicator of conversion efficiency and is shown in the graphs.

### Adoptive cell transfer experiments

CD11b^+^Ly6C^hi^GFP^+^ monocytes were sorted from BM and injected into CD45.1^+^ recipients intravenously (i.v.). Four days after transfer spleen, PB and BM were collected and single cell suspension was prepared. After blocking of Fc receptors using anti-mouse CD16/CD32 (TruStain fcX from Biolegend) cells were labelled with biotin-conjugated anti-CD45.1 antibody, anti-biotin magnetic beads and enriched on LD columns (Miltenyi Biotec) according to the manufacturer's instructions. CD45.1 negative fraction was collected, stained and analysed by flow cytometry. Ly6C^lo^ monocytes (CD11b^+^GFP^+^Ly6C^lo/−^F4/80^lo^CD11c^lo^MHC-II^lo/−^ cells) were quantified in spleen, BM and PB as relative frequency of total donor derived CD45.2^+^CD11b^+^GFP^+^ cells.

### Human monocyte isolation

Human monocytes were isolated from the blood of healthy individuals as approved by the ethical committee of Hannover Medical School. Written consent was obtained before blood collection. Cells were purified using CD16^+^ monocyte isolation kit (Miltenyi Biotec) according to the manufacturer's instruction. CD14^+^ monocytes were retrieved in subsequent purification step from CD16^neg^ fraction using CD14 microbeads.

### Immunohistochemistry and immunofluorescence

Immunohistochemistry, β-galactosidase and immunofluorescence staining in mice were performed with modifications from previous descriptions^37^,^49^. Mice were euthanized; tibiae, spleen, heart, aorta and muscles were isolated and fixed in 4% paraformaldehyde (PFA). Bones were decalcified in 0.5 M EDTA solution at 4 °C for 48 h, cryopreserved in sucrose and embedded in Tissue-tek O.C.T. compound (Sakura, Germany). All the other organs were cryoprotected in sucrose and embedded in Tissue-tek O.C.T. compound without decalcification procedure. β-galactosidase staining was performed at 37 °C on PFA fixed tissues. Slides were counterstained with eosin, mounted in mounting medium and analysed with Olympus IX71 microscope. For immunofluorescence and confocal laser scanning microscopy tissue sections were stained using anti-DLL1 (Biolegend), anti-CD31, anti-CD43 (both from BD Biosciences, Germany), anti-Ly6C (Biolegend) and appropriate fluorescence-conjugated secondary antibodies (Supplementary Table 3). 4,6-Diamidino-2-phenylindole (Invitrogen, Germany) was used for counterstaining of nuclei and slides were mounted in DAKO fluorescence mounting medium (Dako, Denmark). Images were acquired using Leica TCS SP2 AOBS (Leica Microsystems, Germany) confocal microscope or Zeiss Observer Z1 fluorescence microscope (Zeiss, Germany), respectively.

### Intravital microscopy

To visualize monocytes in the microcirculation the cremaster muscle of male *GFP*^*+*^*ctrl* and *GFP*^*+*^*N2*^*ΔMy*^ mice was exposed and transient and adhesive interactions were recorded. To this end an Olympus BX51 microscope equipped with a Hamamatsu 9100-02 EMCCD camera (Hamamatsu Photonics) and a × 40 water-dipping objective was employed. In each cremaster 10 fields of view were recorded for 30 s and the number of adherent cells and the rolling flux (rolling monocytes passing a perpendicular line placed across the observed vessel) from each field were quantified. Subsequent to recordings at baseline, mice were injected via a jugular vein catheter with a single dose of TNF-α (250 ng) and recordings were repeated after 60 min.

### Quantitative real-time PCR analysis

Splenic ECs, as well as splenic or BM monocytes were isolated by cell sorting and total RNA was purified using Nucleospin RNA II kit (Macherey Nagel). After purity and quality check, RNA was transcribed into complementary DNA employing complementary DNA synthesis kit (Invitrogen) according to the manufacturer's instructions. Quantitative real-time PCR was performed using specific primers (Supplementary Table 4) and FastStart Essential DNA Green Master on a LightCycler 96 system from Roche according to the manufacturer's instructions, for normalization. Expression of each specific gene was normalized to expression of *Rps9* and calculated by the comparative CT (2^−ΔΔCT^) method^50^.

### *In vivo* targeting of EC and PCR analysis

Five to 6-weeks-old mice expressing EC-specific inducible *Cre* recombinase and *Cre*-negative littermate controls were treated with 500 μg tamoxifen for 5 consecutive days intraperitoneally (i.p.). Mice were killed after 4 weeks. Efficiency of Cre-dependent gene deletion was monitored by PCR and agarose gel-electrophoresis as described^23^.

### Statistical analysis

Results are expressed as mean±s.e.m.. *N* numbers are biological replicates of experiments performed at least three times unless otherwise indicated. Significance of differences was calculated using unpaired, two-tailed Student's *t*-test with confidence interval of 95%. For comparison of multiple experimental groups one-way analysis of variance (ANOVA) was used and Bonferroni's multiple-comparison test was performed when the overall *P* value was <0.05. Data from intravital microscopy were analysed using two-way ANOVA and Bonferroni's post-test. *P* values of less than 0.05 were considered to be significant.

### Data availability

The authors declare that all the relevant data are available upon request.

## Additional information

**How to cite this article:** Gamrekelashvili, J. *et al.* Regulation of monocyte cell fate by blood vessels mediated by Notch signalling. *Nat. Commun.* 7:12597 doi: 10.1038/ncomms12597 (2016).

## Supplementary Material

Supplementary Information Supplementary Figures 1-10 and Supplementary Tables 1-4
