# Corrigendum: Regulation of monocyte cell fate by blood vessels mediated by Notch signalling

**DOI:** 10.1038/ncomms15486

**Published:** 2017-05-03

**Authors:** Jaba Gamrekelashvili, Roberto Giagnorio, Jasmin Jussofie, Oliver Soehnlein, Johan Duchene, Carlos G. Briseño, Saravana K. Ramasamy, Kashyap Krishnasamy, Anne Limbourg, Christine Häger, Tamar Kapanadze, Chieko Ishifune, Rabea Hinkel, Freddy Radtke, Lothar J. Strobl, Ursula Zimber-Strobl, L. Christian Napp, Johann Bauersachs, Hermann Haller, Koji Yasutomo, Christian Kupatt, Kenneth M. Murphy, Ralf H. Adams, Christian Weber, Florian P. Limbourg

Nature Communications 7: Article number: 12597; DOI: 10.1038/ncomms12597 (2016); Published: 08 31 2016; Updated: 05 03 2017

The authors inadvertently omitted Christine Häger, who was involved in the initial characterization of Notch mutant mice presented in this Article, from the author list and Author contributions statement. These errors have now been corrected in both the PDF and HTML versions of the Article.

