# Supplementary material for: Regulation of monocyte cell fate by blood vessels mediated by Notch signalling
Source: Nat Commun. 2016 Aug 31;7:12597. doi: 10.1038/ncomms12597 (PMC5013671; doi:10.1038/ncomms12597)
Supplement: Supplementary Information — Supplementary Figures 1-10 and Supplementary Tables 1-4 [file ncomms12597-s1.pdf]

Supplementary Figure 1

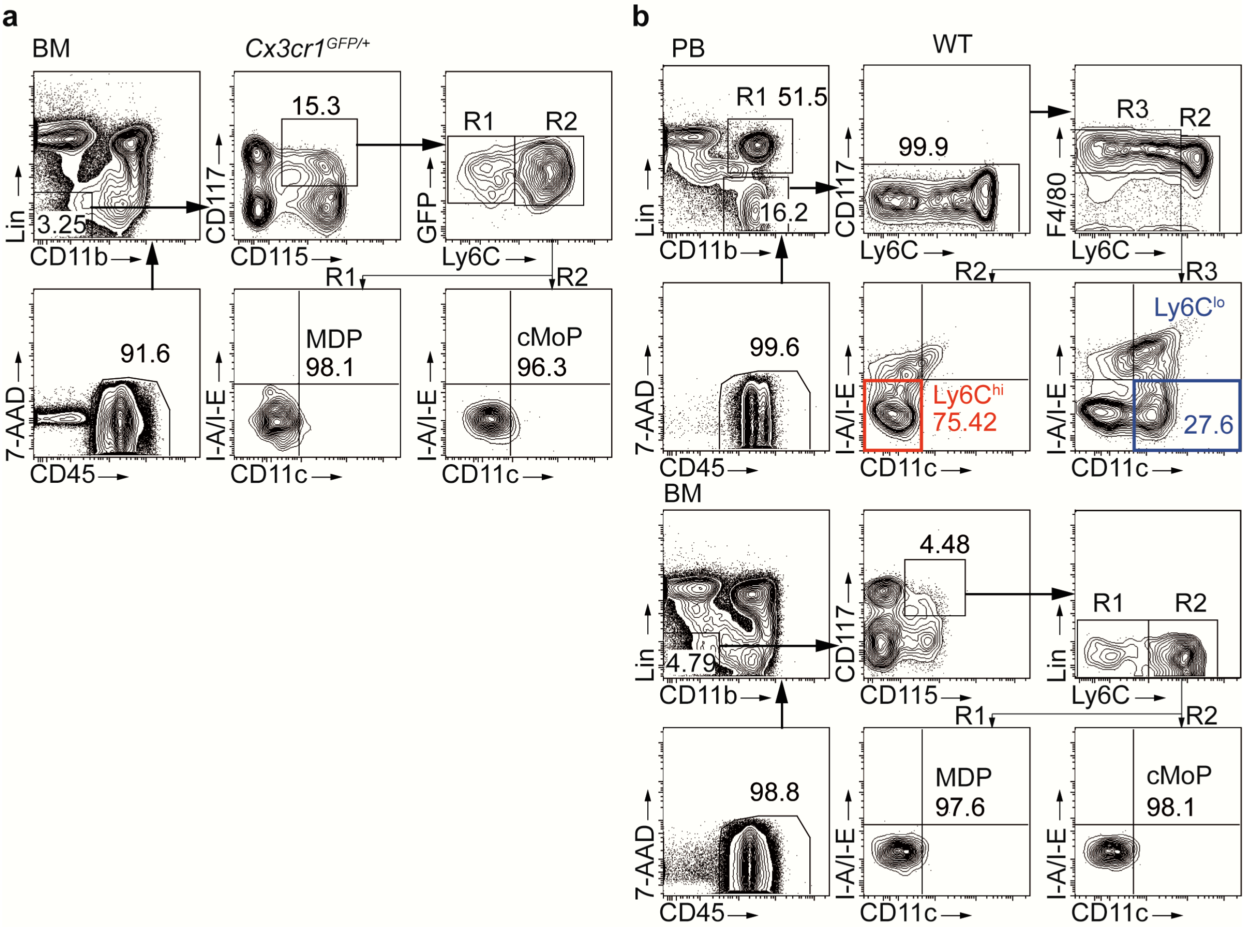

Supplementary Figure 1: Analysis of monocyte subsets and lineage relationships. (**a**) Gating strategy for definition of MDP and cMoP populations in BM of *Cx3cr1*$^{GFP/+}$ mice related to **Fig. 1a**. MDP was defined as CD11c$^-$MHC-II$^-$ cells from live CD45$^+$Lin$^-$CD11b$^-$CD117$^+$CD115$^+$GFP$^+$ population. For cMoP Ly6C$^+$CD11c$^-$MHC-II$^-$ subset was gated from live CD45$^+$Lin$^-$CD11b$^-$CD117$^+$CD115$^+$GFP$^+$ population. (Lin: CD3, CD45R/B220, CD19, NK1.1, Ly6G, CD49b). (**b**) Gating strategy for identification of monocyte and GC (top) or MDP and cMoP (bottom) subpopulations in wild-type (*Cx3cr1*$^{+/+}$) mice.

Supplementary Figure 2

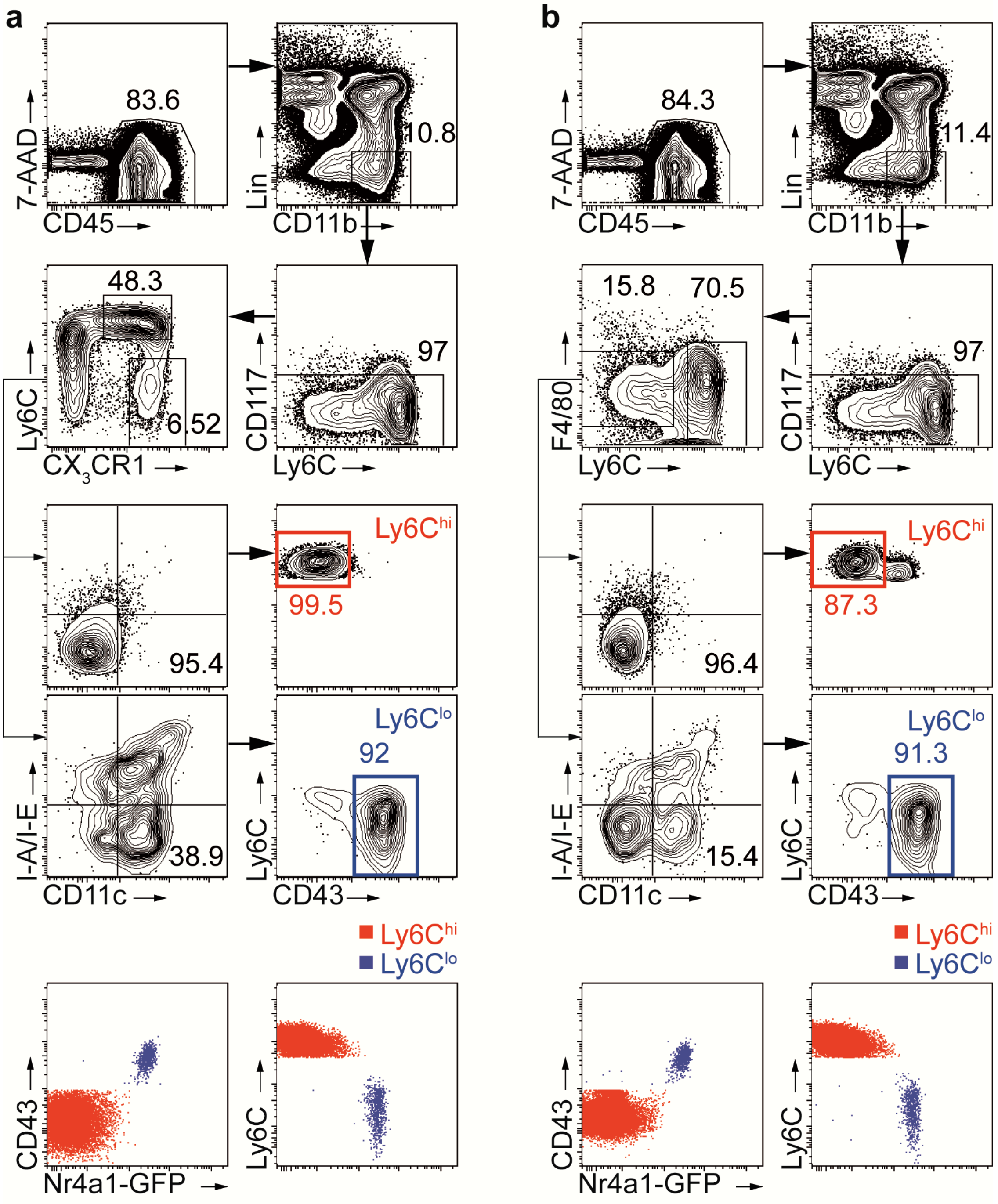

Supplementary Figure 2: Validation of gating strategy in *Nr4a1-GFP* mice and adoptive transfer experiments. (**a**) Flow cytometry plot depicting validation of CX_3CR1 based gating strategy (corresponding to gating in *Cx3cr1*$^{GFP/+}$ mice, in **Fig. 1a**) precisely identifies Nr4a1-GFP$^+$CD43$^+$Ly6C$^{lo/-}$ monocytes in bone marrow. (**b**) Flow cytometry plot depicting validation of CX_3CR1 independent gating strategy (corresponding to gating in wild type mice, in **Supplementary Fig 1b**) precisely defines Nr4a1-GFP$^+$CD43$^+$Ly6C$^{lo/-}$ monocytes.

Supplementary Figure 3

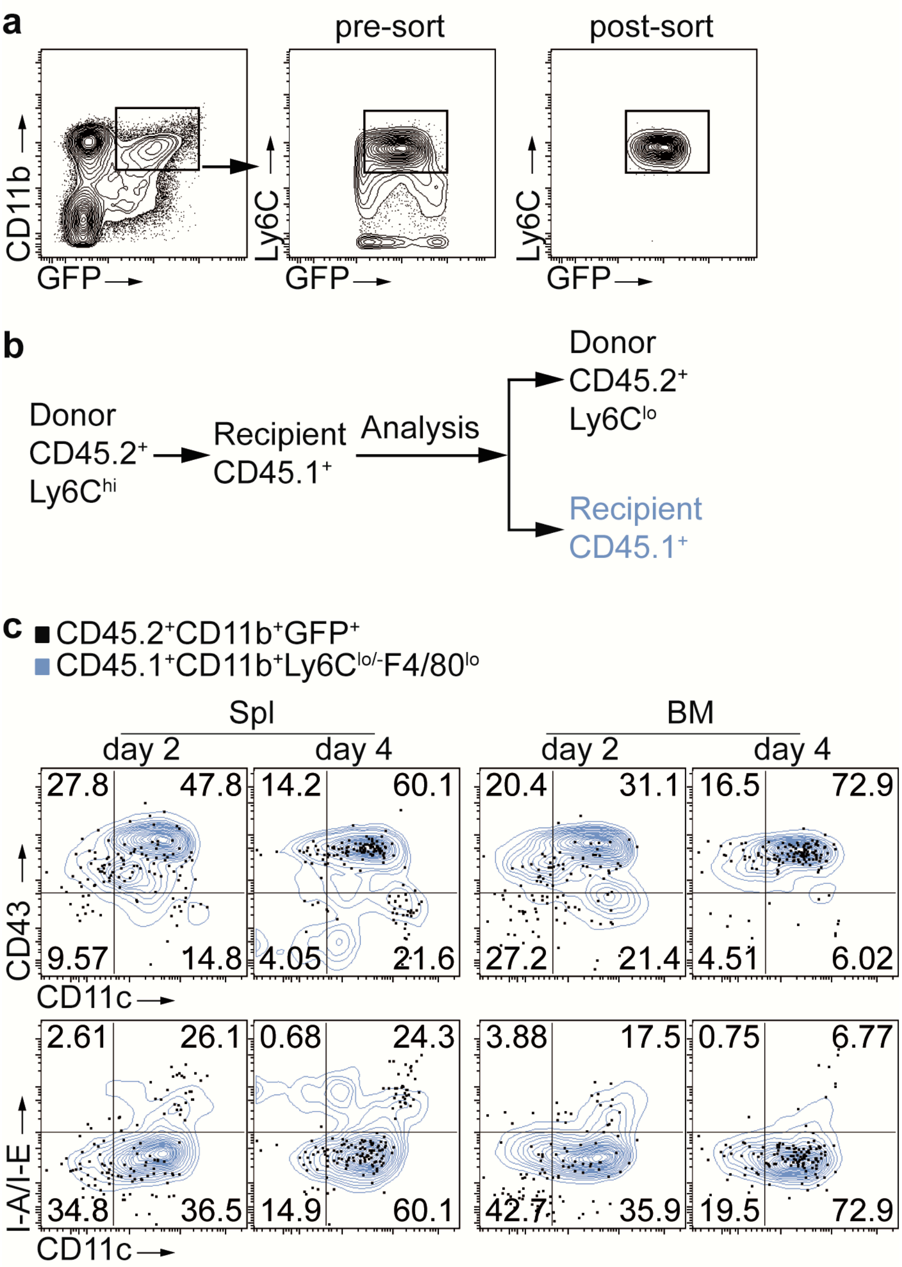

Supplementary Figure 3: Ly6C^{hi} monocyte adoptive transfer experiments. (**a**) Sorting strategy for adoptive transfer of CD11b^{+}GFP^{+}Ly6C^{hi} monocytes. (**b**) Schematic illustration of adoptive transfer and multicolor flow cytometry analysis of Ly6C^{hi} monocytes from GFP^{+}CD45.2^{+} mice into CD45.1^{+} recipients. (**c**) 4th and 5th rows of flow cytometry plots from **Fig. 1d** are shown. Donor CD45.2^{+}CD11b^{+}GFP^{+} cells are black. For comparison CD45.1^{+}CD11b^{+}Ly6C$^{lo/-}$F4/80^{lo} recipient monocytes are depicted in blue (representative of two experiments).

Supplementary Figure 4

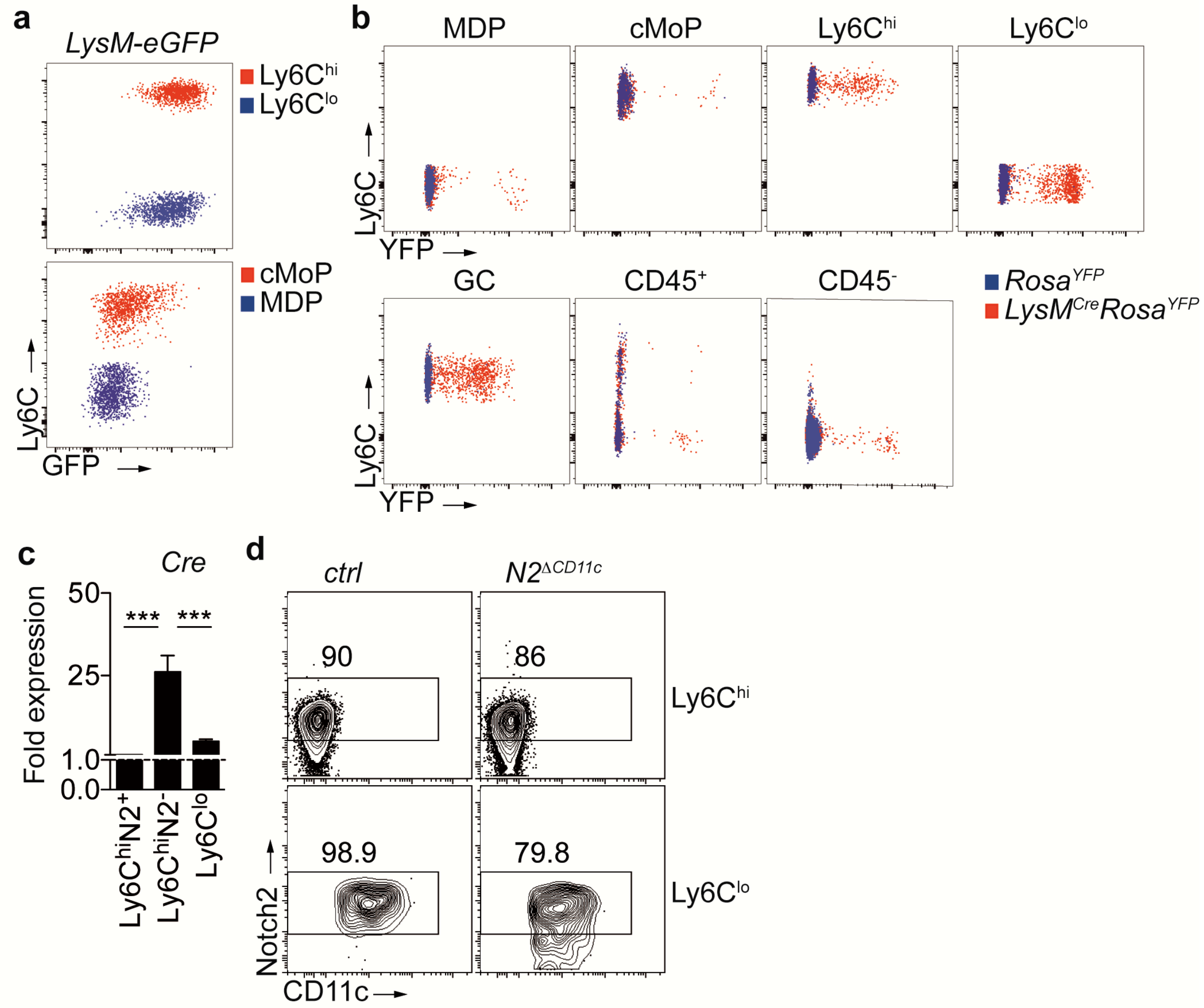

Supplementary Figure 4: Specificity and efficiency of *Cre* expression and *Notch2* targeting. (**a**) Flow cytometry plot showing gradual expression of *LysM-eGFP* in myeloid cell subpopulations from cMoP to Ly6C^{lo} monocytes. (**b**) Flow cytometry plot showing YFP expression in bone marrow of *LysM*^{Cre}*Rosa*^{YFP} or control *Rosa*^{YFP} mice. *LysM*^{Cre} is strongly active in monocytes (Ly6C^{hi} and Ly6C^{lo}) and granulocytes but not in other cell populations. (**c**) Quantitative RT-PCR in sorted bone marrow cells from *GFP*$^{+}$*Notch2*$^{\Delta My}$ mice showing highest expression of *Cre* in N2^{-}Ly6C^{hi} and subsequent reduction in Ly6C^{lo} monocytes (n=6). * *P*<0.05, ** *P*<0.01, *** *P*<0.001; 1way ANOVA with Bonferroni's multiple comparison test. Error bars represent s.e.m. (**d**) Notch2 expression in Ly6C^{hi} and Ly6C^{lo} monocyte subpopulations isolated from BM of *Notch2*$^{\Delta CD11c}$ mice (representative of two experiments). Littermate controls are shown for comparison.

Supplementary Figure 5

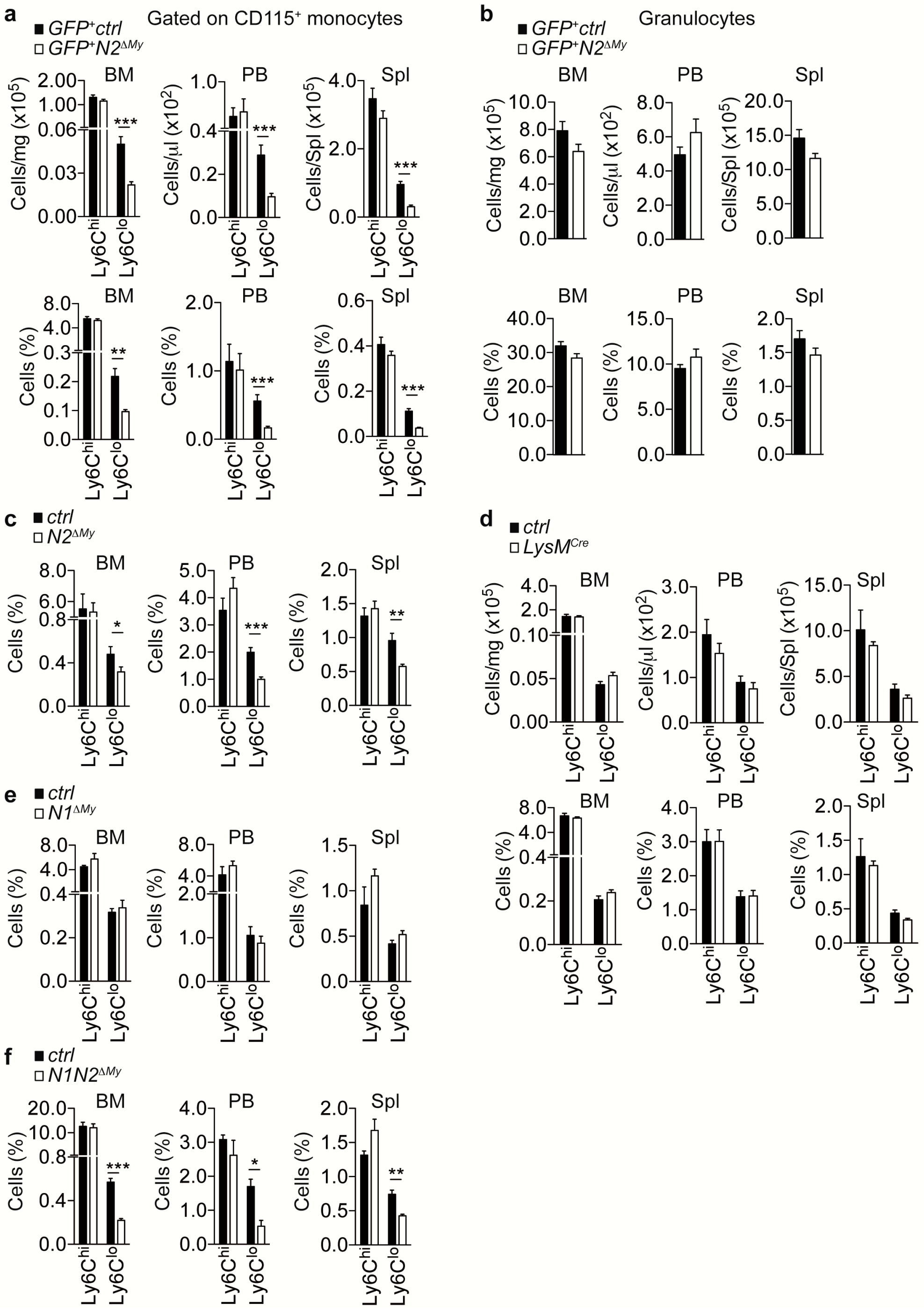

Supplementary Figure 5: Myeloid cell populations in *Notch2* deficient mice. (**a**) Monocyte subpopulation analysis based on CD115+ gating strategy reveals reduction of Ly6C^{lo} but not Ly6C^{hi} monocytes in *GFP^{+}Notch2^{ΔMy}* mice. Data are pooled from three experiments (n=8/11). (**b**) Absolute or relative numbers of granulocytes in *GFP^{+}Notch2^{ΔMy}* mice. Data are pooled from three experiments (n=8/10). (**c**) Relative frequency of monocytes by flow cytometry in *Notch2^{ΔMy}* with wild-type *Cx3cr1* locus. Data are pooled from three experiments (n=9/11). (**d**) Myeloid cell populations in *LysM^{Cre}Notch2$^{+/+}$* and *LysM$^{+/+}$Notch2$^{+/+}$* littermate control mice. Data are from three experiments (n=7/8). (**e**) Myeloid cell population analysis in *N1^{ΔMy}* mice (n=8/9). Data are pooled from three experiments. (**f**) Myeloid cell population analysis in *N1N2^{ΔMy}* mice. Data are pooled from two experiments (n=5/6). (**a-f**) * *P*<0.05, ** *P*<0.01, *** *P*<0.001; Student's t test. Error bars represent s.e.m.

Supplementary Figure 6

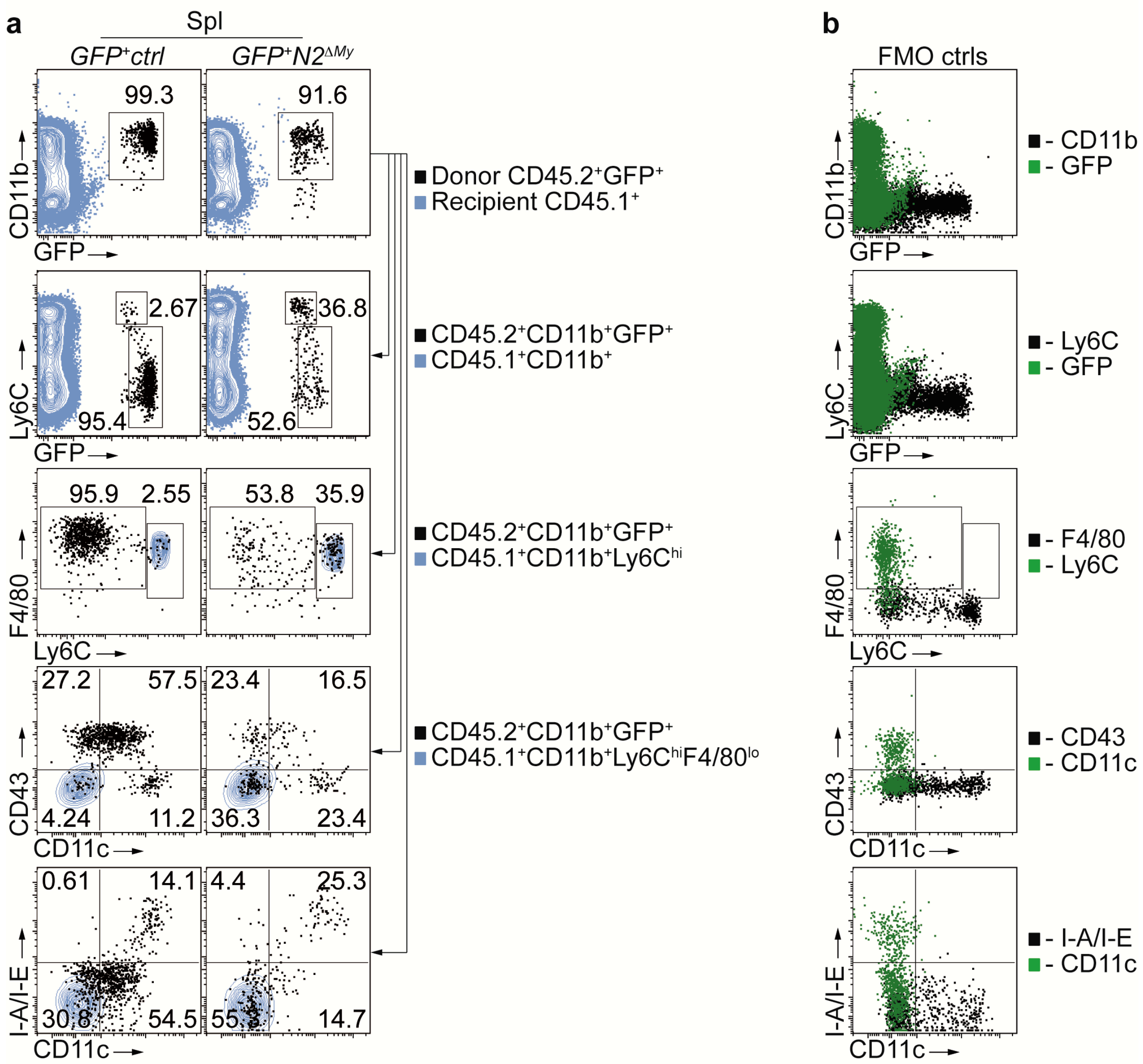

Supplementary Figure 6: Notch2 deficient BM Ly6C^{hi} monocytes show impaired conversion potential *in vivo*. (**a, b**) Flow cytometry (**a**) and corresponding fluorescence minus one controls (FMO ctrls) (**b**) 4 days after adoptive transfer of Ly6C^{hi} monocytes (corresponding to **Fig. 4c**) from control or Notch2 deficient CD45.2$^+$GFP$^+$ donors into CD45.1$^+$ congenic recipients. (**a**) Transferred cells are shown in black and for comparison, recipient CD45.1$^+$ (1st row), CD45.1$^+$CD11b$^+$ (2nd row), CD45.1$^+$CD11b$^+$Ly6C^{hi} (3rd row) cells or CD45.1$^+$CD11b$^+$Ly6C^{hi}F4/80^{lo} monocytes (4th and 5th rows) are depicted in blue.

Supplementary Figure 7

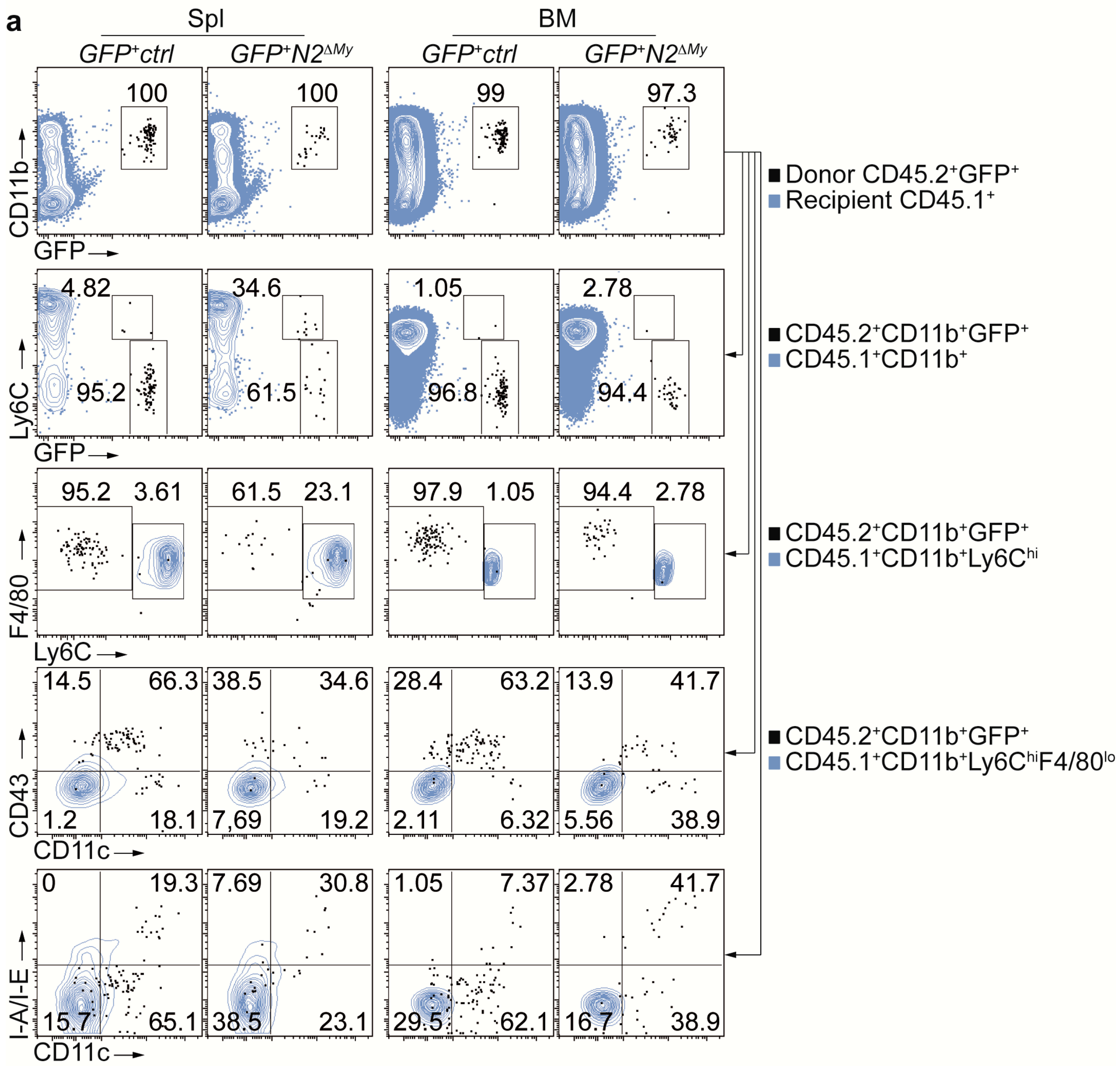

Supplementary Figure 7: Notch2 deficient peripheral Ly6C^{hi} monocytes show impaired conversion potential *in vivo*. (**a**) Flow cytometry plots 4 days after adoptive transfer of pooled splenic and peripheral blood Ly6C^{hi} monocytes from control or Notch2 deficient CD45.2^{+}GFP^{+} donors into CD45.1^{+} congenic recipients. Transferred cells are shown in black and for comparison, recipient CD45.1^{+} (1st row), CD45.1^{+}CD11b^{+} (2nd row), CD45.1^{+}CD11b^{+}Ly6C^{hi} (3rd row) cells or CD45.1^{+}CD11b^{+}Ly6C^{hi}F4/80^{lo} monocytes (4th to 5th rows) are depicted in blue (representative of two experiments).

Supplementary Figure 8

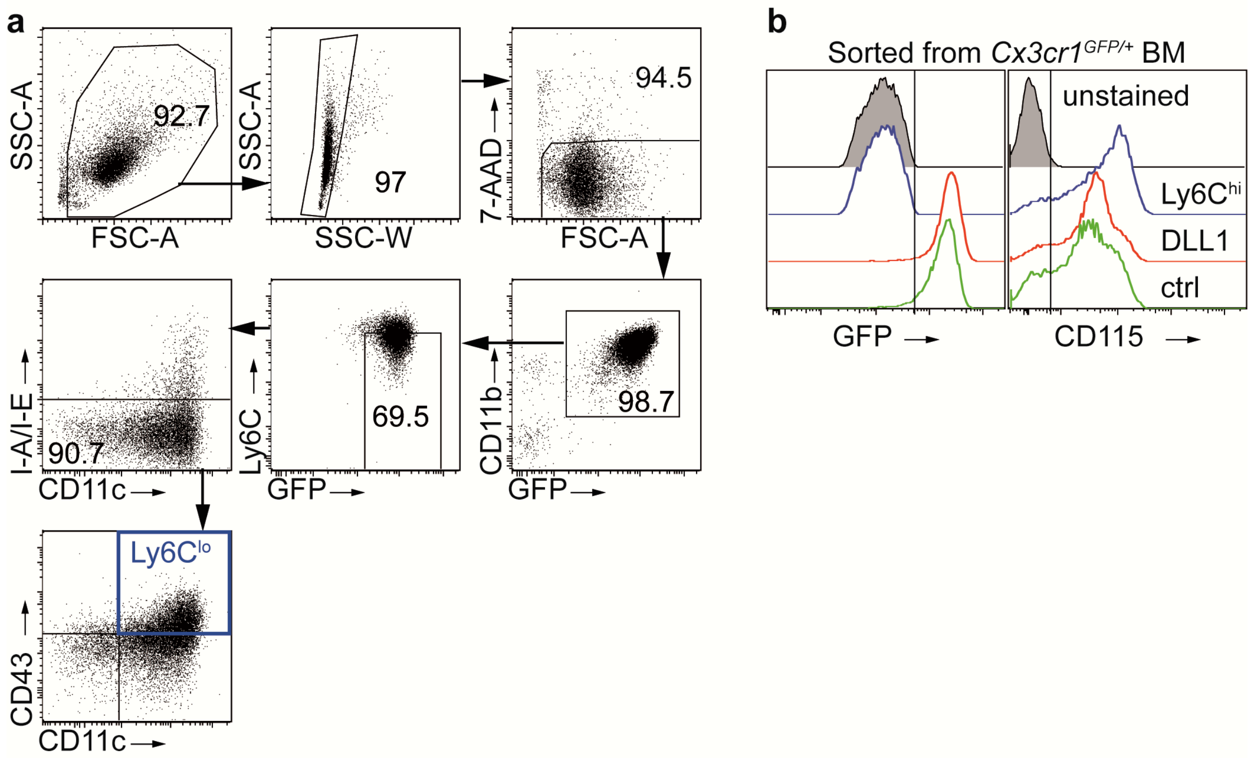

Supplementary Figure 8: Notch ligand DLL1 mediates monocyte conversion *in vitro*. (**a**) Gating strategy for definition and quantification of Ly6C^{lo} monocyte-like cells *in vitro*. Ly6C^{lo} monocyte-like cells (CD11b^{+}GFP^{+}Ly6C$^{lo/-}$CD11c^{lo}CD43^{+}MHC-II$^{lo/-}$) are calculated as a percentage of live CD11b^{+}GFP^{+} cells. (**b**) Flow cytometry plot showing expression of CD115 and upregulation of GFP on *in vitro* converted Ly6C^{lo} monocyte-like cells. *Ex vivo* isolated GFP^{+}Ly6C^{hi} BM monocytes served as a staining control.

Supplementary Figure 9

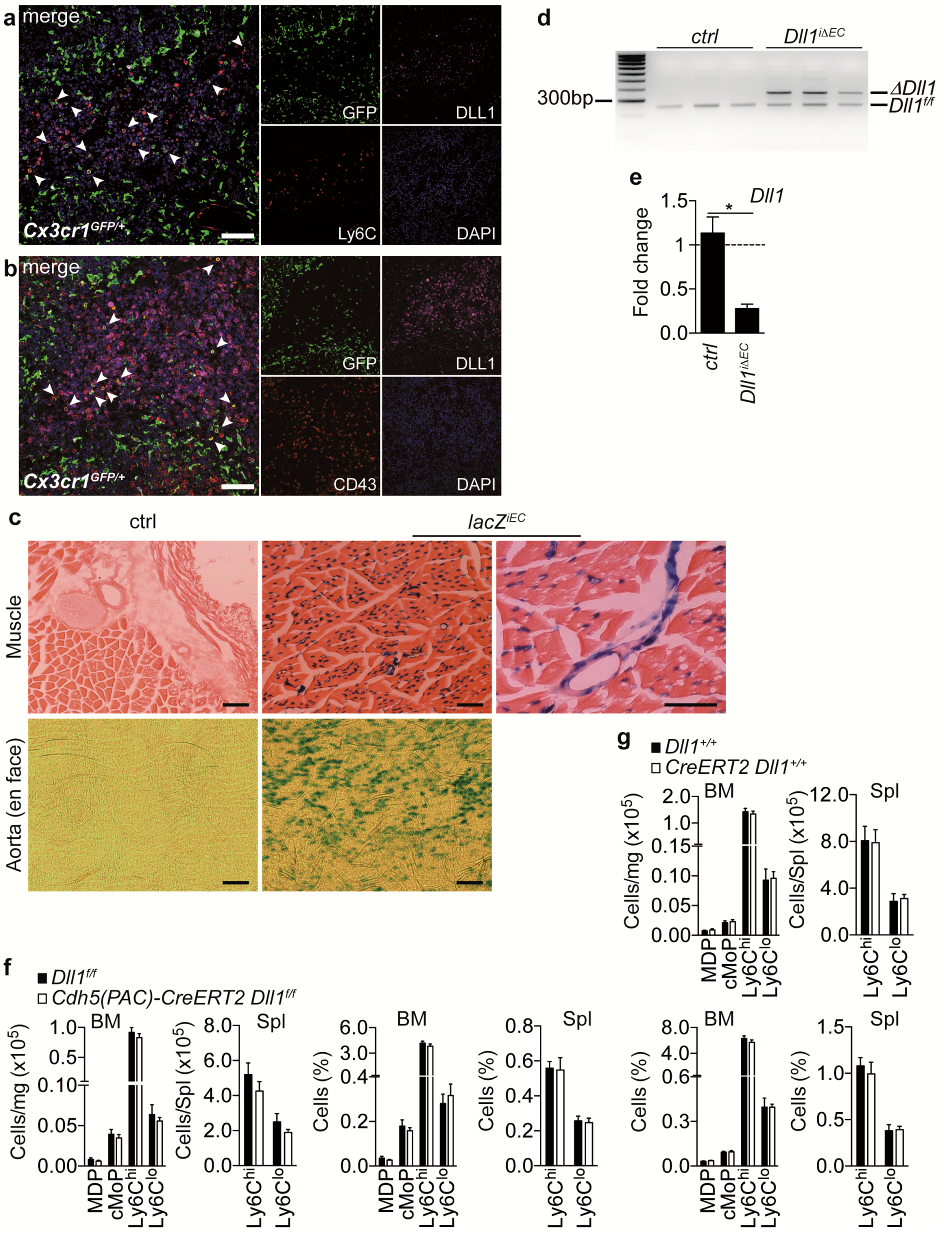

Supplementary Figure 9: *In vivo* targeting of endothelial cells in mice. b(**c**) Specific β-galactosidase activity in capillaries and large vessels of peripheral muscle (top) and in aortic EC (en face preparations (bottom)) after treatment of *lacZ*^{iEC} or control mice with tamoxifen; scale bar 100µm. Image is a representative of two (muscle) or three (aorta) experiments. (**d**) PCR for floxed or recombined locus of *Dll1* from heart (left), lung (middle) and peripheral muscle (right). Results are from one experiment representative of 4 experiments. (**e**) Quantitative RT-PCR analysis of *Dll1* expression in sorted ECs from control or *Dll1*$^{i\Delta EC}$ mice. Pooled from three experiments (n=3). (**f**) Myeloid cell population analysis in *Dll1*$^{+/+}$ or *CreERT2 Dll1*$^{+/+}$ mice after tamoxifen treatment showing no influence of tamoxifen on Ly6C^{lo} monocyte development. Data are pooled from two experiments (n=6/7). (**g**) Cell population analysis in *Cdh5(PAC)-CreERT2 Dll1*$^{f/f}$ mice showing no influence of *Cdh5(PAC)-CreERT2* expression on Ly6C^{lo} monocyte development. Data are pooled from three experiments (n=6/8). (**e-g**) * *P*<0.05, ** *P*<0.01, *** *P*<0.001; Student's t test. Error bars represent s.e.m.

Supplementary Figure 10

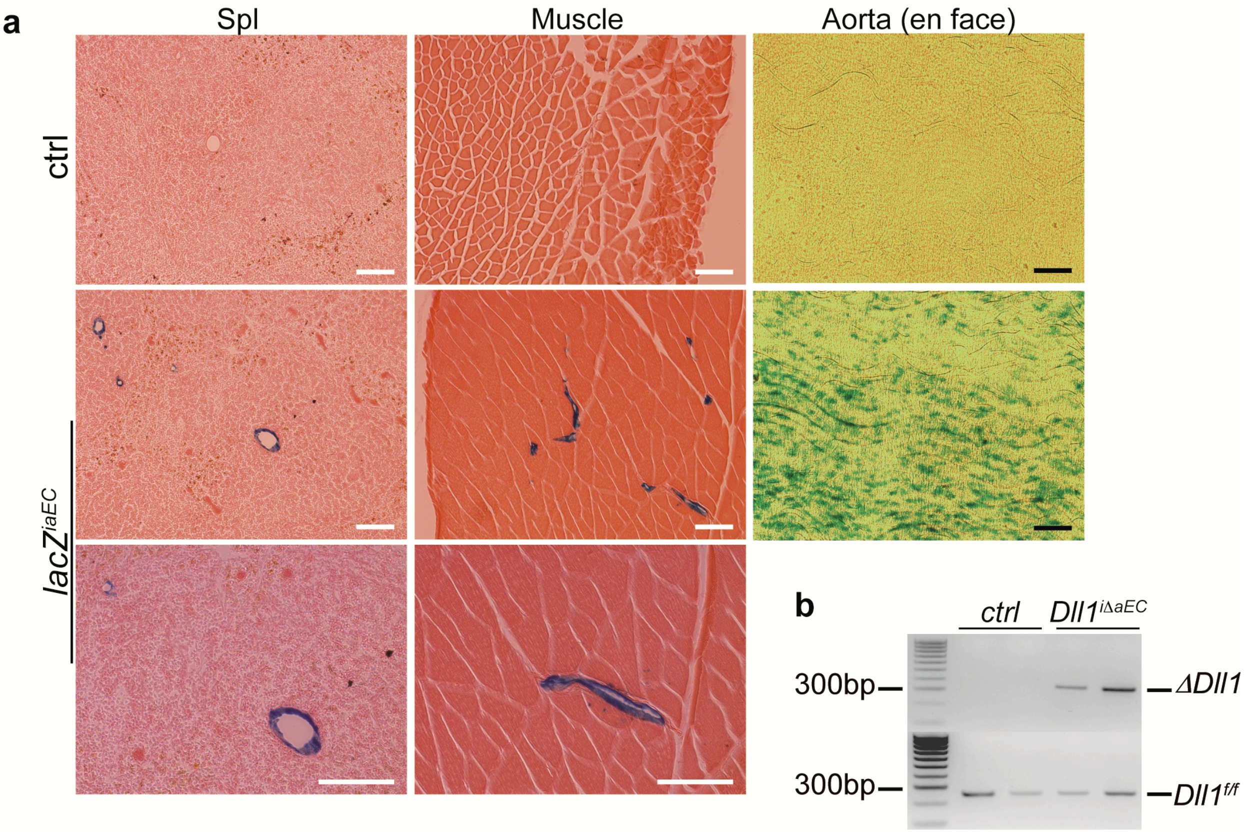

Supplementary Figure 10: *In vivo* targeting of arterial endothelial cells in mice. (**a**) β-galactosidase staining demonstrates specific staining in central arteries of splenic follicles, arteries of peripheral muscle and aorta in *lacZ*^{iaEC} mice. Scale bars 100µm. Results are from one experiment representative of two independent experiments. (**b**) PCR for floxed or recombined *Dll1* locus (*ΔDll1*) from aortas (n=2).

Supplementary Table 1: Surface phenotype signatures for identification of distinct myeloid populations *in vivo*

Population	Phenotype
MDP	Lin^{-}CD117^{+}CD11b^{-}CD115^{+}CX$_{3}$CR1^{+}Ly6C^{-}F4/80^{-}CD11c^{-}MHC-II^{-}<br>or<br>Lin^{-}CD117^{+}CD11b^{-}CD115^{+}Ly6C^{-}F4/80^{-}CD11c^{-}MHC-II^{-}
cMoP	Lin^{-}CD117^{+}CD11b^{-}CD115^{+}CX$_{3}$CR1^{+}Ly6C^{hi}F4/80^{-}CD11c^{-}MHC-II^{-}<br>or<br>Lin^{-}CD117^{+}CD11b^{-}CD115^{+}Ly6C^{hi}F4/80^{-}CD11c^{-}MHC-II^{-}
Ly6C^{hi}	Lin^{-}CD117^{-}CD11b^{+}CX$_{3}$CR1^{lo}Ly6C^{hi}F4/80$^{lo/-}$CD11c^{-}MHC-II$^{lo/-}$CD43^{-}<br>or<br>Lin^{-}CD117^{-}CD11b^{+}Ly6C^{hi}F4/80$^{lo/-}$CD11c^{-}MHC-II$^{lo/-}$CD43^{-}
Ly6C^{lo}	Lin^{-}CD117^{-}CD11b^{+}CX$_{3}$CR1^{hi}Ly6C$^{lo/-}$F4/80^{lo}CD11c^{lo}MHC-II$^{lo/-}$CD43^{+}<br>or<br>Lin^{-}CD117^{-}CD11b^{+}Ly6C$^{lo/-}$F4/80^{lo}CD11c^{lo}MHC-II$^{lo/-}$CD43^{+}
GC	Lin^{+}CD11b^{+}CX$_{3}$CR1^{-}Ly6C^{lo}<br>or<br>Lin^{+}CD11b^{+}Ly6C^{lo}
Atypical cells (Ly6C^{lo})	Lin^{-}CD117^{-}CD11b^{+}CX$_{3}$CR1^{hi}Ly6C$^{lo/-}$F4/80^{lo}CD11c^{-}MHC-II^{hi}CD43^{-}<br>or<br>Lin^{-}CD117^{-}CD11b^{+}Ly6C$^{lo/-}$F4/80^{lo}CD11c^{-}MHC-II^{hi}CD43^{-}

Lin: CD3, CD45R/B220, CD19, NK1.1, Ly6G, CD49b

Supplementary Table 2: Mouse models used in the study

Abbreviations	Mouse description	Mouse background
CD45.1^{+}	B6.SJL-*Ptprc*^{a}*Pepc*^{b}/BoyJ	*B6*
GFP^{+} *ctrl*<br>*GFP*$^{+}$ *N2*$^{\Delta My}$	*LysM*$^{+/+}$ *Notch2*$^{lox/lox}$ *Cx3cr1*$^{GFP/+}$<br>*LysM*^{Cre} *Notch2*$^{lox/lox}$ *Cx3cr1*$^{GFP/+}$	B6<br>*B6*
Ctrl<br>*N2*$^{\Delta My}$	*LysM*$^{+/+}$ *Notch2*$^{lox/lox}$<br>*LysM*^{Cre} *Notch2*$^{lox/lox}$	*B6*<br>*B6*
Ctrl<br>*N1*$^{\Delta My}$	*LysM*$^{+/+}$ *Notch1*$^{lox/lox}$<br>*LysM*^{Cre} *Notch1*$^{lox/lox}$	*B6*<br>*B6*
Ctrl<br>*N1N2*$^{\Delta My}$	*LysM*$^{+/+}$ *Notch1*$^{lox/lox}$ *Notch2*$^{lox/lox}$<br>*LysM*^{Cre} *Notch1*$^{lox/lox}$ *Notch2*$^{lox/lox}$	*B6*<br>*B6*
wt<br>*Dll1*$^{+/lacZ}$	*Dll1*$^{+/+}$<br>*Dll1*$^{+/lacZ}$	*129*<br>*129*
Ctrl<br>*lacZ*^{iEC}<br><br>*lacZ*^{iaEC}	*Gt(ROSA)26Sor*<br>*Cdh5(PAC)-CreERT2*<br>*Gt(ROSA)26Sor*<br>*Bmx(PAC)-CreERT2*<br>*Gt(ROSA)26Sor*	*B6*<br>*B6*<br><br>*B6*
Ctrl<br>*Dll1*$^{i\Delta EC}$<br>*Dll1*$^{i\Delta aEC}$	*Dll1*$^{lox/lox}$<br>*Cdh5(PAC)-CreERT2 Dll1*$^{lox/lox}$<br>*Bmx(PAC)-CreERT2 Dll1*$^{lox/lox}$	*Mixed, B6;129*<br>*Mixed, B6;129*<br>*Mixed, B6;129*
Ctrl<br>*Dll4*$^{i\Delta EC}$	*Dll4*$^{lox/lox}$<br>*Cdh5(PAC)-CreERT2 Dll4*$^{lox/lox}$	*Mixed, B6;CD1*<br>*Mixed, B6;CD1*
Dll1$^{+/+}$<br>*CreERT2 Dll1*$^{+/+}$	*Dll1*$^{+/+}$<br>*Cdh5(PAC)-CreERT2 Dll1*$^{+/+}$	*Mixed, B6;129*<br>*Mixed, B6;129*
Ctrl<br>*LysM*^{Cre}	*LysM*$^{+/+}$ *Notch2*$^{+/+}$<br>*LysM*^{Cre} *Notch2*$^{+/+}$	*B6*<br>*B6*
Nr4a1-GFP	*Nr4a1-EGFP/Cre*	*B6*
LysM-eGFP	*LysM-EGFP*	*B6*
Rosa^{YFP}<br>*LysM*^{Cre}*;Rosa*^{YFP}	*LysM*$^{+/+}$ *Gt(ROSA)YFP26Sor*<br>*LysM*^{Cre} *Gt(ROSA)YFP26Sor*	*B6*<br>*B6*
GFP^{+} *ctrl*<br><br>*GFP*$^{+}$ *N1N2*$^{\Delta CD11c}$	*CD11c*^{Cre} *Notch1*$^{lox/+}$ *Notch2*$^{lox/+}$<br>*Cx3cr1*$^{GFP/+}$<br>*CD11c*^{Cre} *Notch1*$^{lox/lox}$ *Notch2*$^{lox/lox}$<br>*Cx3cr1*$^{GFP/+}$	*B6*<br><br>*B6*
Ctrl<br>*N2*$^{\Delta CD11c}$	*CD11c*$^{+/+}$ *Notch2*$^{lox/lox}$<br>*CD11c*^{Cre} *Notch2*$^{lox/lox}$	*B6*<br>*B6*

Supplementary Table 3: Antibodies and fluorescence dyes for flow cytometry and immunofluorescence used in the study

Antibody/dye	Clone	Dilution	Company
Anti-CD3ε	145-2C11	1:100	BioLegend
Anti-CD49b	DX5	1:400	BioLegend
Anti-CD45R/B220	RA3-6B2	1:400	BioLegend
Anti-Ly6G	1A8	1:400	BioLegend
Anti-CD19	1D3	1:400	BD Pharmingen
Anti-NK1.1	PK136	1:400	BioLegend
Anti-CD117	2B8	1:100	BioLegend
Anti-CD115	AFS98	1:100	BioLegend
Anti-CD11b	M1/70	1:400	BioLegend
Anti-Ly6C	HK1.4	1:2800	BioLegend
Anti-F4/80	BM8	1:100	BioLegend
Anti-CD11c	N418	1:400	BioLegend
Anti-I-A/I-E	M5/114.15.2	1:400	BioLegend
Anti-CD43	S7	1:400	BD Pharmingen
Anti-CD45	30-F11	1:200	BioLegend
Anti-CD45.1	A20	1:100	BioLegend
Anti-CD45.2	104	1:200	BioLegend
Anti-CD144	11D4.1	1:100	BD Pharmingen
Anti-CX_3CR1	SA011F11	1:200	BioLegend
Anti-CD11a	M17/4	1:200	BioLegend
Anti-CCR2	475301	1:100	R&D
Anti-Notch2	HMN2-35	1:100	BioLegend
Anti-CD16/CD32	93	1:200	BioLegend
Anti-CD31	Mec13.3	1:400	BD Pharmingen
Streptavidin PE-Dazzle594		1:400	BioLegend
Annexin V		1:50	BioLegend
7AAD		1:100	BioLegend
Propidium Iodide		1:12000	Sigma
Anti-GFP		1:300	Acris
Anti-DLL1	HMD1-3	1:100	BioLegend
Streptavidin Cy3		1:400	BioLegend
Anti-rabbit IgG-FITC		1:200	Jackson ImmunoResearch
DAPI		1:5000	Invitrogen

Supplementary Table 4: Primers for QRT-PCR

Gene	Primers
Notch2	Forward: AGTGTCAGAGGCCAGCAAGAAGAA<br>Reverse: TGATTGTCGTCCATCAGAGCACCA
Notch1	Forward: TGGAGGTCTCAGTGGCTATAA<br>Reverse: ATTCTGGCATGGGTTAGAAAGA
Hey2	Forward: TGAAGCGCCCTTGTGAGGAA<br>Reverse: TTGTAGCGTGCCCAGGGTAA
Hes1	Forward: CCGGACAAACCAAAGACGGC<br>Reverse: GGAATGCCGGGAGCTATCTTTCT
Dll1	Forward: TCCGATTCCCCTTCGGCTTC<br>Reverse: TGGGTTTTCTGTTGCGAGGT
Nr4a1	Forward: AGCTTGGGTGTTGATGTTCC<br>Reverse: AATGCGATTCTGCAGCTCTT
Pparg	Forward: AGGGCGATCTTGACAGGAA<br>Reverse: CACCTCTTTGCTCTGCTCCT
Pou2f2	Forward: TGCACATGGAGAAGGAAGTG<br>Reverse: AGCTTGGGACAATGGTAAGG
Slfn5	Forward: AGGTCGAACGATTCTGCTGT<br>Reverse: TCTGAGGGAAACTGGAAAGG
Ccr2	Forward: CCTTGGGAATGAGTAACTGTGTGAT<br>Reverse: ATGGAGAGATACCTTCGGAACTTCT
Cx3cr1	Forward: GCAGAAGTTCCCTTCCCATC<br>Reverse: GGACAGGAAGATGGTTCCAA
